# Global genome decompaction leads to stochastic activation of gene expression as a first step toward fate commitment in human hematopoietic cells

**DOI:** 10.1371/journal.pbio.3001849

**Published:** 2022-10-26

**Authors:** Romuald Parmentier, Laëtitia Racine, Alice Moussy, Sophie Chantalat, Ravi Sudharshan, Nan Papili Gao, Daniel Stockholm, Guillaume Corre, Geneviève Fourel, Jean-François Deleuze, Rudiyanto Gunawan, Andras Paldi

**Affiliations:** 1 École Pratique des Hautes Études, PSL Research University, St-Antoine Research Center, Inserm U938, AP-HP, SIRIC CURAMUS, Paris, France; 2 Centre National de Recherche en Génomique Humaine, Evry, France; 3 Department of Chemical and Biological Engineering, University, Buffalo, New York, United States of America; 4 Institute for Chemical and Bioengineering, ETH Zurich, Zurich, Switzerland; 5 Genethon, Evry, France; 6 Laboratory of Biology and Modelling of the Cell, University of Lyon, ENS de Lyon, University of Claude Bernard, CNRS UMR 5239, Inserm U1210, Lyon, France; 7 Centre Blaise Pascal, ENS de Lyon, Lyon, France; Institute for Systems Biology, UNITED STATES

## Abstract

When human cord blood–derived CD34+ cells are induced to differentiate, they undergo rapid and dynamic morphological and molecular transformations that are critical for fate commitment. In particular, the cells pass through a transitory phase known as “multilineage-primed” state. These cells are characterized by a mixed gene expression profile, different in each cell, with the coexpression of many genes characteristic for concurrent cell lineages. The aim of our study is to understand the mechanisms of the establishment and the exit from this transitory state. We investigated this issue using single-cell RNA sequencing and ATAC-seq. Two phases were detected. The first phase is a rapid and global chromatin decompaction that makes most of the gene promoters in the genome accessible for transcription. It results 24 h later in enhanced and pervasive transcription of the genome leading to the concomitant increase in the cell-to-cell variability of transcriptional profiles. The second phase is the exit from the multilineage-primed phase marked by a slow chromatin closure and a subsequent overall down-regulation of gene transcription. This process is selective and results in the emergence of coherent expression profiles corresponding to distinct cell subpopulations. The typical time scale of these events spans 48 to 72 h. These observations suggest that the nonspecificity of genome decompaction is the condition for the generation of a highly variable multilineage expression profile. The nonspecific phase is followed by specific regulatory actions that stabilize and maintain the activity of key genes, while the rest of the genome becomes repressed again by the chromatin recompaction. Thus, the initiation of differentiation is reminiscent of a constrained optimization process that associates the spontaneous generation of gene expression diversity to subsequent regulatory actions that maintain the activity of some genes, while the rest of the genome sinks back to the repressive closed chromatin state.

## Abstract 2

A study of the mechanisms of fate commitment in human hematopoietic cells, using single-cell RNA-seq and ATAC-seq, reveals that early in differentiation there is non-specific chromatin decompaction which creates the opportunity for stochastic gene activation; over time the chromatin closes and the initial burst in gene expression is refined into a coherent profile corresponding to cell subpopulations.

## Introduction

Understanding the process of cell differentiation that generates functionally and morphologically different cells with distinct gene expression profiles is one of the major challenges in biology. The way cell differentiation is conceptualized has changed during the last years [1]. Initially, cell differentiation was considered as a predetermined sequence of molecular and cellular events programmed by the genome. In this classical cause-and-effect paradigm, the new phenotype is induced by the action of specific signals that activate specific genes resulting in a linear deterministic process of cell fate determination and phenotypic differentiation [2,3]. The idea of linear causation has been progressively undermined by the large amount of data provided by the various “omics” approaches that raised the urgent need for generalizable principles [4]. The introduction of the conceptual arsenal of the dynamical complex system’s field can potentially satisfy this need [4] and provide an example how mathematics and physics can stimulate thinking in biology [5]. It is now generally accepted that molecular interactions within the cell, including gene transcription and translation, are fundamentally stochastic [6,7]. First considered as a simple “noise” perturbing the neatly functioning of the deterministic regulatory pathways, now it is becoming likely that the molecular variations are part of the system and play an essential biological role [8]. This view is further reinforced by the demonstration that molecular fluctuations are not only ubiquitous, but the cell is unable to suppress them by specifically dedicated mechanisms [9]. The conceptual framework of the complex dynamical systems allows the incorporation of molecular stochasticity and the resulting nonlinear dynamics in the explanatory scheme [10]. Importantly, the fundamental role of molecular stochasticity in cell differentiation was conjectured long time ago by Kupiec [11–13]. He proposed that cell differentiation can be viewed as a process of selective stabilization of gene expression profiles generated by spontaneous stochastic variation of gene transcription. The initial theory has been further developed [14,15] and now supported by a large body of experimental observations [16].

Frequently considered as a paradigm of cell differentiation in general, hematopoietic cells are widely used as experimental model to study fate commitment. The differentiation of the hematopoietic cells is frequently represented as a series of binary fate decisions under the action of key instructive factors inducing specific changes in the cell and leading to progressively decreasing capacity of self-renewal, proliferation and lineage potential [17,18]. Such a strict hierarchical process must imply tight regulation of the expression of key genes. A number of genes that play a key role in the process and the core gene regulatory network (GRN) of hematopoiesis have been identified [19,20]. The early ideas [3] about gene regulation acting linearly during differentiation evolved toward the dynamic system view and the conceptual framework of the complex dynamical systems is now applied to the study of the hematopoietic differentiation also [21]. The concept of stochasticity has also appeared early in the study of hematopoietic differentiation, thanks to the pioneering work by Till [22]. Single-cell gene expression studies added a new layer to the general picture. They demonstrated that soon after their stimulation for differentiation, multipotent CD34+ cells go through a phase of disordered gene expression called “multilineage-primed” phase characterized by concomitant expression of genes typical for alternative lineages [23–26]. More recent studies confirmed that hematopoietic stem cells (HSCs) gradually acquire lineage characteristics along multiple directions without passing through discrete hierarchically organized and demarcated progenitor populations [27] and that lineage-restricted cells emerge directly from a “continuum of low-primed undifferentiated hematopoietic stem and progenitor cells” [27]. It has been shown that this phase is accompanied by instabilities and fluctuations of the cell transcriptome, morphology, and dynamic cell behavior essentially during the first 2 to 3 cell cycles [26,28]. How this quasi-random gene expression pattern is generated remains unclear. Indeed, it is hardly possible to imagine that a different strictly regulated hierarchical processes targeting specific genes could generate a unique mixed gene expression pattern in each cell and subsequently make them to converge to the same defined profile. In order to determine how such a response is produced, we investigated the early chromatin and transcriptional changes during the short initial period of time when the critical fate decision is initiated in CD34+ cells.

To do this, we correlated the dynamic changes of the transcription profiles determined by single-cell RNA sequencing (scRNA-seq) at different time points during the 96-h period following their stimulation with the chromatin profiles during the same period as determined by bulk and single-cell ATAC sequencing (scATAC-seq). The data revealed that a rapid and global nonspecific chromatin decompaction precedes the global up-regulation of gene expression by an unusually long lag of 24 h. Specific regulatory actions may come at the next stage to stabilize and maintain the activity of a subset of genes that allow the cell to better thrive in the changing environment. The remaining part of the genome becomes repressed again as a consequence of the chromatin recompaction.

## Results

**Fig 1:**
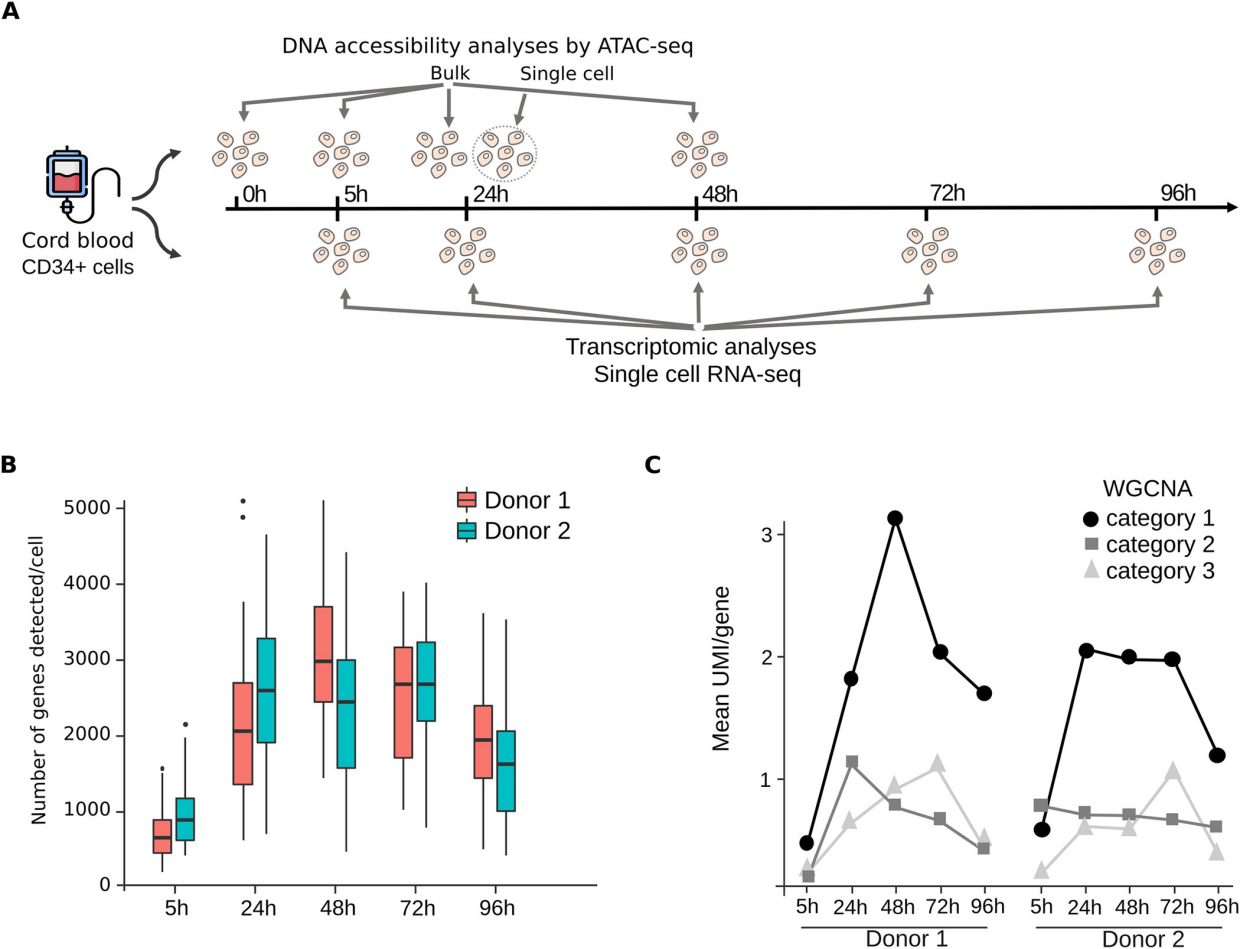
Experimental strategy and global gene expression dynamics. (**A**) CD34+ cells were isolated from human cord blood and cultured in serum-free medium with early acting cytokines. scRNA-seq was used to analyze transcription at 5 h, 24 h, 48 h, 72 h, and 96 h. Concomitantly, at 0 h, 5 h, 24 h, and 48 h, 5,000 living cells were collected for “bulk” ATAC-seq analysis of the DNA accessibility. The 24-h time point was analyzed by scATAC-seq also. (**B**) Number of detected genes per cell with scRNA-seq. Two donors were analyzed separately, both showed similar dynamics. The exact numbers are indicated in the Results section. Note the rapid increase in the number of genes expressed per cell between 5 h and 24 h and the slow decrease after a plateau between 24 h and 72 h. (**C**) WGCNA reveals groups of genes with similar dynamic patterns in the average mRNA expression in donor1 and donor2. Details about WGNCA are given in **Materials and methods.** Note that category 1 reproduces the best overall dynamic pattern observed for genes showing detectable expression in single cells in (**B**). Category 1 = 5,194 genes (donor1) and 5,518 genes (donor2), category 2 = 3,977 genes (donor1) and 2,602 (donor2), category 3 = 1,089 genes (donor1) and 609 genes (donor2). (Numerical values available in scRNA-seq repository GSE156734: “GSE156734_Spread_MARSseq_data_all_filters_20200728.csv”) scATAC-seq, single-cell ATAC sequencing; scRNA-seq, single-cell RNA sequencing; WGCNA, weighted correlation network analysis.

Our experimental strategy (Fig 1A) was as follows. First, we evaluated the progression of the human CD34+ cord blood cells toward defined fates after cytokine stimulation using scRNA-seq. This approach allowed us to assess quantitatively the phenotypic heterogeneity and identify subpopulations at each time point within the time window defined by our previous study [26]. Then, we investigated the genome-scale changes of the chromatin structure using whole-cell population-level ATAC-seq (referred to as bulk ATAC-seq). scATAC-seq was used at a critical time point to confirm the conclusions. Finally, we analyzed the data to determine how global chromatin changes are related to global transcription changes. As the hematopoietic system is a very well-studied experimental model and the key individual elements are well known, we focused our analysis on the less known global tendencies rather that individual genes and chromatin elements.

### An initial transcription burst precedes stable expression profiles

We used human CD34+ cells isolated from the cord blood of 2 healthy donors and cultured in the presence of early acting cytokines as described previously [26]. We performed massively parallel single-cell RNA sequencing (MARS-seq; see **Materials and methods**) at different time points. The cells were isolated randomly from the CD34+ fraction to ensure a correct statistical representation of the whole population without any preconceived ideas on the cell phenotypes and categories. Based on our previous studies [26] showing that before cytokine stimulation the CD34+ cells have very low transcriptional activity, we set 5 h poststimulation as the starting point, followed by sampling at 24 h, 48 h, 72 h, and 96 h after the cells were cultured in the presence of cytokines. The advantage to use MARS-seq is the high sensibility of the method, which allows the reliable detection of low numbers mRNA molecules per gene in each cell. To ensure the reliable quantification, we used unique molecular identifier (UMI)-marked synthetic mRNAs (details about quality control of the results are shown in **S1 Table**). As a result, we were able to obtain high-resolution quantitative transcription profiles for individual cells. To avoid technical variation, the cells of the 2 donors were processed parallelly and sequenced on the same flow cell. However, the batch correction procedure tends to remove some relevant information too; hence, we chose to represent the results separately for each donor. Separating the donors allowed us to assess the similarities of the global tendencies while conserving the potentially important inter- and intraindividual heterogeneity of the temporal progression.

The analysis revealed important features in global gene expression dynamics (**Fig 1B and 1C**). Following stimulation, the transcriptome underwent rapid and substantial quantitative and qualitative changes. Both the number of expressed genes per cell and the number of mRNA molecules per gene increased substantially. The average number of expressed genes detected per cell at 5 h was only 512 +/− 243 in donor1. This number increased to 1,693 +/− 813 at 24 h and 2,543 +/− 751 at 48 h, but then decreased to 2,014 +/− 714 at 72 h and to 1,612 +/− 613 at 96 h. The tendency for donor2 were very identical (5 h– 760 genes +/− 297, at 24 h– 2,298 genes +/− 822, at 48 h– 2,036 genes +/− 809, at 72 h– 2,217 genes +/− 612, and at 96 h– 1,420 genes +/− 630). The increase in global transcription activity is very rapid, it occurred between 5 h and 24 h, suggesting that the cells expand their repertoire of transcribed genes (**Fig 1B**) as the initial phase of the fate decision process. During this phase, each cell expresses a unique collection of transcripts. After 72 h, the number of genes expressed per cell started to decrease, coinciding with the time when the first signs of lineage-specific transcriptional changes appear [26]. While the initial burst in mRNA levels is the likely consequence of increased transcription activity, it is worth mentioning that the MARS-seq protocol allows the detection of steady-state mRNA levels only. Since these levels are strongly dependent both on the transcription and degradation rates of the mRNA molecules, our observations only partially reflect the actual transcriptional activity of the genes.

We used weighted correlation network analysis (WGCNA) of gene expression to investigate whether group of genes with distinct correlated dynamics can be identified. Similar categories of genes with highly correlated mean expression patterns over time were defined in both donors (**Fig 1C**). The 3 largest categories together sum up to more than 10,200 genes for donor1 and 8,700 genes for donor2. Although with slightly different dynamics, all gene categories display an initial increase followed by a decrease, pointing to a genome-wide phenomenon. Thus, the CD34+ cells of both donors responded to cytokine stimulation in a similar way, with a strong, but transient, gene up-regulation both in terms of gene and transcripts numbers.

**Fig 2:**
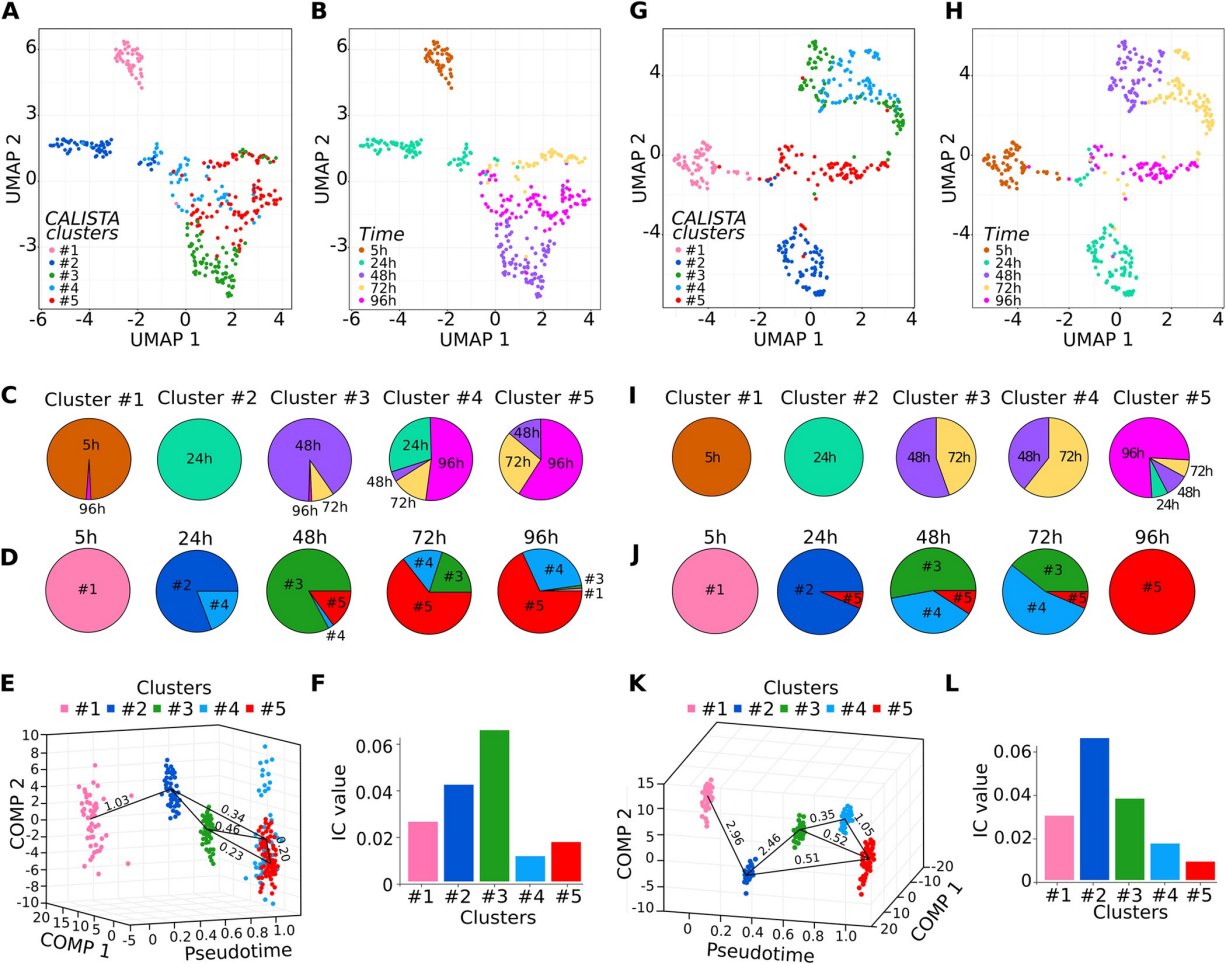
Evolution of transcriptome profiles after cell stimulation. The 2 donors are presented separately: donor1 on the left panels (**A** to **F**) and donor2 on the right (**G** to **L**). (**A**, **B** and **G**, **H**) UMAP visualization of the single-cell RNA data. Each point represents a single cell. The cells belonging to the same cluster identified by CALISTA are color coded on (**A** and **G**). On (**B** and **H**), the cells are colored according to the time point they were collected. Note the good agreement with the separate UMAP groups. (**C** and **I**) Composition of the CALISTA clusters according to the time point the cells were collected. CALISTA clustered the cells according to their transcription profile similarity. Note that the heterogeneity of the cell population increases in time and concomitantly to the transcription changes as the individual cells progressed at different pace. (**D** and **J**) Clusters composition of the groups of cells collected at the different time points. Note the relative homogeneity of the groups at early time points and their gradual diversification at later time points. (**E** and **K**) Time progression of the cells through different transcription states as determined by CALISTA. The transition edges with the cluster distances are shown. They suggest that different simultaneous trajectories are possible. The cluster color code is the same as in (**D** and **J**). Comp1 and Comp2 are the first 2 axes of the PCA used by CALISTA for dimensionality reduction. (**F** and **L**) The index for critical transitions (I_c_) calculated separately for each cluster. Note that in both donors, the index reaches the maximum value in clusters #2 and #3, indicating a phase of critical transition. (Numerical values available in scRNA-seq repository **GSE156734**: “GSE156734_CALISTA_results_d1csv.gz” “GSE156734_Spread_MARSseq_data_all_filters_20200728.csv” “GSE156734_CALISTA_results_d2.csv.gz”) CALISTA, Clustering And Lineage Inference in Single-cell Transcriptional Analysis; PCA, principal component analysis; UMAP, Uniform Manifold Approximation and Projection.

To investigate the structure of the cell population, we first visualized the data using the usual dimension reduction method, Uniform Manifold Approximation and Projection (UMAP) (**Fig 2A, 2B, 2G and 2H**). For both donors, the cells collected at different time points clustered separately, suggesting a clear time progression in gene expression. In order to identify cell clusters with similar gene expression patterns, characterize their lineage progression and the possible trajectories of the cells, we analyzed our data using Clustering And Lineage Inference in Single-cell Transcriptional Analysis (CALISTA). This method is specifically dedicated to the analysis of single-cell RNA data [29]. On the basis of the 2-state stochastic model of gene transcription [30], CALISTA identifies cell clusters. The algorithm calculates and assigns a likelihood value to each cell that reflects the joint probability of its gene expression pattern and mRNA levels. On the basis of the 200 most variable genes (**S2 Table**), for both donors, CALISTA identified 5 single-cell clusters (**Fig 2A and 2G**). These clusters almost perfectly overlapped with the 5 visually distinguishable groups of cells on UMAP. For both donors, clusters #1 and #2 were essentially composed of cells isolated at 5 h and 24 h, respectively (**Fig 2C and 2I**). Clusters #3, #4, and #5 were mixed containing cells collected at 24 h, 48 h, 72 h, and 96 h. Correspondingly, when the cells were grouped according to the time point they were collected, we observed that some cells reached the profile corresponding to clusters #4 or #5 as early as 48 h, while others needed 96 h to do so (**Fig 2D and 2J**). This suggests that the gene expression burst was initiated immediately after cytokine stimulation and then the cells progressed at their own pace and gradually became desynchronized. It is worth to remind that the time-lapse observations [26] showed that the first cell cycle after stimulation lasts on average 56 h. Therefore, it is likely that the initial global increase in gene expression, the establishment of the mixed quasi-random expression profile and the beginning of the specification of distinct profiles occur during the same single cell cycle.

CALISTA also calculated “cluster distances” between each pair of clusters. This metrics is based on the maximum difference in the cumulative likelihood values of the gene expression distribution [31]. A small cluster distance makes the transition between them more likely than a large one. Thus, these distances helped to visualize the likely sequences of the lineage progression. The 2 graphs show that the cells of both donors display similar overall lineage trajectories (**Fig 2E and 2K**). Importantly, the close distances between clusters #3, #4, and #5 make likely that different simultaneous trajectories are possible and a cell can reach any of these clusters through different pathways or switch between them, as suggested by the previously reported time-lapse observations [26].

Cell differentiation can be conceptualized as a transition between 2 stable states of the underlying gene expression network (GRN) configurations [32]. GRNs can be described using the tools designed for the study of dynamic complex systems. The rise and decrease of global transcription intensity in our cells coincided with the period of multilineage-primed state and is reminiscent of a transition between 2 states of complex dynamic systems. Therefore, in order to determine how far the different cell clusters are from the critical transition point, we used the “index for critical transitions” (I_c_) [32], a simple metric of dynamic systems adapted to gene expression networks. The evolution of this index indicates an upcoming state transition of the system independently of the exact mechanism of the transition by simply considering the variance between and within the elements of the system, i.e., the cells. To determine the I_c_, we calculated the gene–gene Pearson correlations between all pairs of gene vectors (R(g_n_,g_m_)) and the cell–cell correlation between all pairs of cell state vectors (R(c_i_,c_j_)). The analysis was performed separately for each cluster and each donor. Only the correlations with a Pearson coefficient higher than 0.70 were taken into account. The I_c_ is calculated as the ratio between the average of all R(g_n_,g_m_)-s and R(c_i_,c_j_)-s [32]. The results shown on **Fig 2F and 2L** indicate that in both donors, the I_c_ sharply increased toward a maximum between 24 h and 48 h, followed by a gradual decrease by 72 h to 96 h. This dynamic is a typical hallmark of a critical transition between 24 h and 48 h and indicates that these cells are close to or going through a critical transition point.

In order to determine if specific genes are involved in the massive transcription burst observed during the initiation of cell fate transition, we performed a comparative gene ontology (GO) analysis of the genes expressed in the cell clusters. Based on the dynamical network biomarker method [33], we used for this analysis the list of genes for which the pairwise gene–gene correlation score was greater than 0.70. The top “molecular function” GO categories (*p* < 0.01) were compared between the clusters (**S3 Table** and **S1 Fig**). The analysis showed similar enriched GO terms among clusters for both donor1 and donor2. Cluster #1 is characterized essentially with broad-spectrum terms associated to translation, transcription activities, and cellular interactions. These categories constitute a common base for the differentiation between the clusters. Clusters #2, #3, and #4 showed the greatest variety of enriched GO terms, ranging from nucleotide synthesis to metabolic activities, but with no apparent cell type–related functions. Finally, in cluster #5, GO terms pointing to erythroid lineage–related functions emerged (see **S3 Table** for GO terms enrichment statistics), suggesting that these cells were progressing in their lineage commitment as described earlier [26].

**Fig 3:**
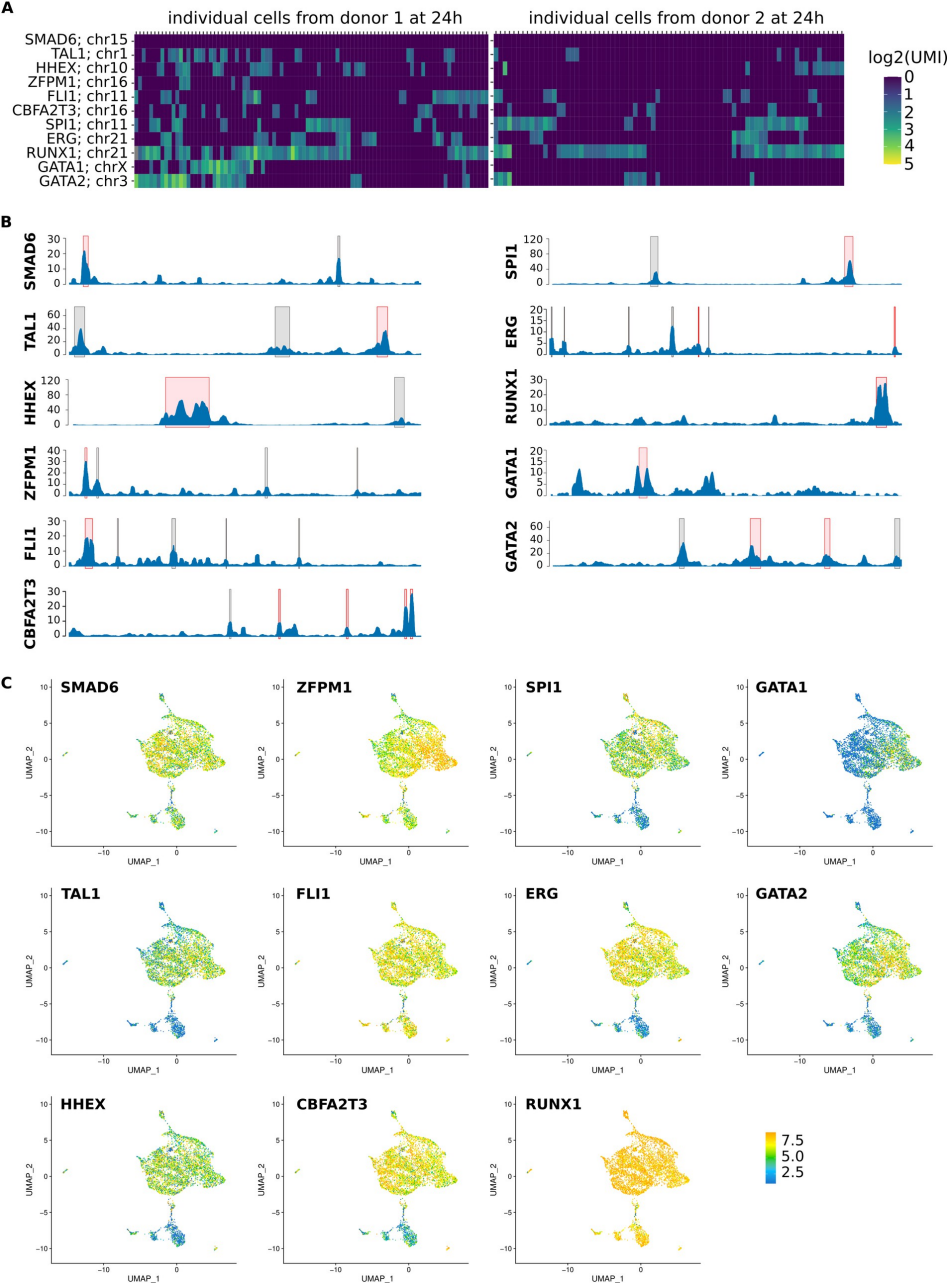
Single-cell transcript levels and accessibility of 11 hematopoietic TFs. (**A**) Heat maps representing the single-cell transcript levels in individual cells for donor1 (left) and donor2 (right) at 24 h. Each raw represents a single gene. The gene name abbreviations are indicated for both panels on the left. Each column represents a single cell. Note the heterogeneity of transcript levels for each gene and in each cell. All the time points (5 h, 24 h, 48 h, and 96 h) are shown on the S2 **Fig**. (**B**) ATAC-seq accessibility profiles of the same 11 genes (in the same order) as determined by the “bulk” approach at the 24-h time point. (All the other time points are shown on the S2 **Fig**). The red boxes on the average profile indicate the accessible promoter-located peaks. Note that every gene has accessible promoters irrespective of the expression state shown on (**A**). The size of the peaks is indicated in normalized “read counts”. The genes are not drawn to scale. (**C**) UMAP representation of the accessibility of the 11 hematopoietic TF-coding genes as determined by scATAC-seq at 24 h. The same UMAP projection (also shown on Fig 5D) was colored as a function of the log2 number of integrations within the whole gene (the color code is on the right). Note the substantial cell-to-cell and gene-to-gene heterogeneity of the accessibility. scATAC-seq, single-cell ATAC sequencing; TF, transcription factor; UMAP, Uniform Manifold Approximation and Projection.

With the exception of the cluster #5, the GO analysis could not detect coherent functional categories of coexpressed genes. To validate the results of this analysis in a more explicit way, we examined the expression of 11 transcription factor (TF)-coding genes known to be specifically involved in the definition of hematopoietic progenitor functions typical for early stages of differentiation [20]. All of them showed high cell-to-cell variation and any of them was expressed in every cell (**Figs 3A** and S2). Overall, these genes followed the general temporal pattern seen for the WGCNA category 1 genes. At 5 h, only sporadic TF expression was detected in the minority of the cells. At 24 h and 48 h, both the number of expressing cells and the mRNA molecules increased followed by a decrease at 72 h and 96 h (**Figs 3A** and S2). The highest expression was seen for RUNX1, GATA2, and SPI1, 3 TFs considered as “pioneer” factors. At 5 h in both donors, only 10% to 40% of cells showed some expression of these 3 genes (S2 **Fig**). At 48 h, they were expressed in up to 80% of the cells. Others, like FLI1 or GATA1, showed no expression at 5 h and sporadic expression was detected later stages. Their mosaic expression did not contribute to the classification of the cells into subgroups (clusters). Recently, Weinreb and colleagues [34] also found that the list of expressed genes defining the subtypes in HSPCs is almost entirely composed by highly expressed and highly variable genes, and only marginally enriched in TFs. These observations are at odds with the usual idea that TFs in general and pioneer TFs more specifically play a key role in initiating gene expression changes by chromatin opening and directly stimulating the transcription of their target genes [35,36]. Our observations on the TF-coding genes revealed that they followed the same dynamic increase after the stimulation of the cells as all other genes. Their role, including the pioneer TFs such as GATA1, GATA2, SPI1, or RUNX1, in the initial transcription burst appears therefore minor for the simple reason that they are expressed at a very low level and in the minority of the cells only. Thus, the transcription burst is likely to be initiated by other causes than TFs.

### Chromatin decondensation as a nonspecific response to cell stimulation

To get a better insight in the first steps of the process, we investigated the chromatin structure changes. We used ATAC-seq to determine the DNA accessibility in the CD34+ cells [37]. First, bulk ATAC-seq was used to establish the temporal dynamics of the chromatin and identify the global systemic changes in chromatin structure [38]. Then, scATAC-seq at the most critical time point was used to confirm the observations.

**Fig 4:**
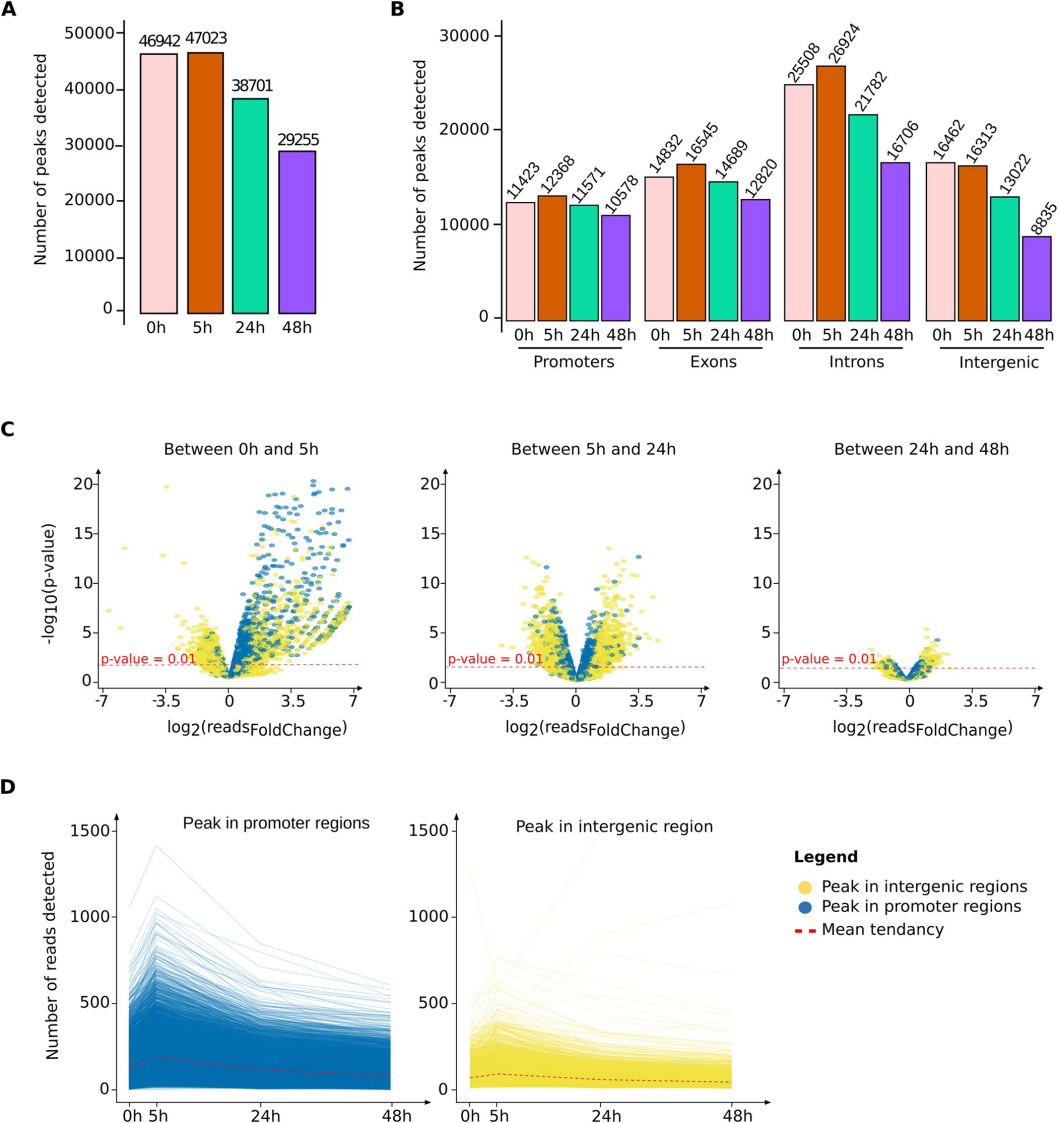
Rapid decompaction and slow recompaction of the chromatin as detected by ATAC-seq. (**A**) Total number of accessible regions (peaks) detected in all 3 independent donors at 4 different time points as determined by “bulk” ATAC-seq. Note the highest number of peaks is seen at 5 h. (**B**) Distribution of the peaks in (**A**) in different genomic elements. A single peak may count for 2 categories if it spans 2 elements (promoter and first exon, for example) with the exception of the intergenic category defined by the exclusion of all the others. (**C**) Volcano plot representation of the quantitative changes of the peaks detected at 2 consecutive time points (0 h and 5 h on the left plot; 5 h and 24 h in the middle; and 24 h and 48 h on the right). The plots show how the size of the peaks that are detected at both time points change. Each point represents the difference between the size of the same peak (as log2 of the number of reads) and the *p*-value of the change. Peaks in promoter regions are highlighted in blue and in intergenic regions in yellow. The extent of the changes is calculated as log2 read-counts (on the horizontal axis). The log10 *p*-value is given in the vertical axis. The threshold of 0.01 is indicated by a red spotted line in each plot. Note the significant increase in size (accessibility) between 0 h and 5 h and the decreasing total number of changes after 24 h. (**D**) Evolution of the ATAC peaks in promoters (blue, left panel) and intergenic regions (yellow, right panel). Each line represents the evolution of the population average DNA accessibility at the same single genomic location between the time points. There are 8,972 peaks mapped to promoters and 6,171 peaks mapped to intergenic positions. The size of each ATAC peak is plotted for every time point. Each line connects the points corresponding to the ATAC peaks detected at the same genomic position. Only the peaks detected at each time point are represented. The red spotted line indicates the mean tendency. Note the general tendency to pass through a maximum at 5 h. (A minority of peaks displayed different evolution; they are shown on **S5 Fig**). (Numerical values available in bulk ATAC-seq repository **GSE156733**: “GSE156733_readCount_00h_Xvivo.txt.gz”; “GSE156733_readCount_05h_Xvivo.txt.gz”; “GSE156733_readCount_24h_Xvivo.txt.gz”; “GSE156733_readCount_48h_Xvivo.txt.gz”. “GSE156733_peaks_intersection_00h_Xvivo_ann.csv.gz”; “GSE156733_peaks_intersection_05h_MP_ann.csv.gz”; “GSE156733_peaks_intersection_24h_MP_ann.csv.gz”; “GSE156733_peaks_intersection_48h_MP_ann.csv.gz”. Numerical values available in bulk ATAC-seq repository **GSE156733**: *“*GSE156733_DEseq2_results_05h_MP_vs_00h_Xvivo.txt.gz”; *“*GSE156733_DEseq2_results_24h_MP_vs_05h_MP.txt.gz”; “GSE156733_DEseq2_results_48h_MP_vs_24h_MP.txt.gz”; “GSE156733_readCount_00h_Xvivo.txt.gz”; “GSE156733_readCount_05h_Xvivo.txt.gz”; “GSE156733_readCount_24h_Xvivo.txt.gz”; “GSE156733_readCount_48h_Xvivo.txt.gz”; “GSE156733_peaks_intersection_00h_Xvivo_ann.csv.gz”; “GSE156733_peaks_intersection_05h_MP_ann.csv.gz”; “GSE156733_peaks_intersection_24h_MP_ann.csv.gz”; “GSE156733_peaks_intersection_48h_MP_ann.csv.gz”).

Bulk ATAC-seq analysis was performed on 5,000 cells of 3 independent donors at 4 time points (0 h, 5 h, 24 h, and 48 h after cell stimulation). Relevant accessible regions were identified using a stringent filter based on the reproducible detection of accessibility in all 3 donors [39] (see **S4 Table** for donor-related information). We found that a large number of DNA regions were already accessible at 0 h (**Fig 4A**) around the time the cells encounter the cytokines. Their number slightly increased between 0 h and 5 h around the transcription start sites/promoters, in the introns and exons, but not in the intergenic regions, then decreased gradually at relatively slow rate over the next 48 h (**Fig 4B**). The time-dependent decrease in the number of ATAC-seq peaks varied with their genomic location. While the number of peaks in distal intergenic regions was halved between 5 h and 48 h, the decrease in the other locations was less important (**Fig 4B**). In particular, the number of accessible promoter regions decreased by only 15% between 0 h and 48 h. These changes indicate a rapid and global reorganization of the chromatin structure.

Next, we have analyzed how individual peaks changed over time. First, we estimated the size of the peaks that were detected at least at 2 consecutive time points. As a proxy for the size of a peak, we used the number of sequenced reads (read counts) that define it. The increase or decrease in read counts for a peak in the same genomic position and between 2 consecutive time points was used to assess the tendency of the chromatin to open or close. We calculated the log-fold changes of the number of reads of each peak for time intervals and the associated *p*-values and represented them as volcano plots (**Fig 4C**). We observed a tendency for the peaks already present at 0 h to further increase in accessibility by 5 h, in particular peaks located in the promoter regions (blue dots in **Fig 4C**). Between 5 h and 24 h, approximately equal proportions of peaks increased or decreased. Between 24 h and 48 h, the size of the persisting remained stable. Overall, our ATAC-seq analysis shows that the chromatin is already relaxed at 0 h and undergo further changes in accessibility during the first 48 h. First, new genomic elements become accessible and others, already open, become more accessible during the first 5 h. Then, the trend is reversed: Both the number and size of ATAC-seq peaks decreased between 5 h and 24 h. The latter trend was maintained, albeit at a lesser degree, between 24 h and 48 h. This analysis provided a quantitative assessment of the major trends between 2 time points in term of peak numbers, but it gave no information on the evolution dynamics of individual peaks. Therefore, we plotted the size of each peak at each time. This representation gave a precise account of the changes at each peak and at the same time provided an overview of the general tendency. On **Fig 4D**, we represented the peaks detected in promoters and intergenic regions at all 4 time points. In the intergenic region, only 27% of the peaks are detected at all time points. In both cases, the size of the peaks increased between 0 h and 5 h and gradually decreased between 5 h and 48 h (**Fig 4D**). The peaks that displayed more complex dynamics are represented on **S3 Fig**; either they appeared later than 5 h or disappeared completely at some stage. However, in both categories, the general tendency to decrease remained the same.

Then, we focused our analysis on the accessibility profiles of the 11 hematopoietic TF-coding genes that showed heterogenous cell-to-cell expression [20] (**Figs 3B** and **S4**). The ATAC profiles showed that at least one of the promoters of these genes were already accessible at 0 h and remained so at 24 h and 48 h (**Figs 3B** and **S4**).

Next, we analyzed the accessibility of the transcription factor binding site (TFBS) motifs. We observed that many of the TFBSs of factors known hematopoietic TFs, such as RUNX1, ERG, PU.1, and FLI1, were highly accessible at 0 h and remained accessible at a similar level up to 48 h (**S5 Fig** and **S5 Table**). The easy access to a large variety of promoters may explain how the mixed multilineage-primed transcription profile can emerge in these cells. We also noted that CTCF (CCCTC-binding factor) binding sites were detected more than 5 times more frequently in the accessible regions than expected on the basis of their frequency in the genome. Indeed, CTCF is known to play a key role of chromatin remodeling and loop formation in general [40] and more specifically in the hematopoietic lineage [41].

**Fig 5:**
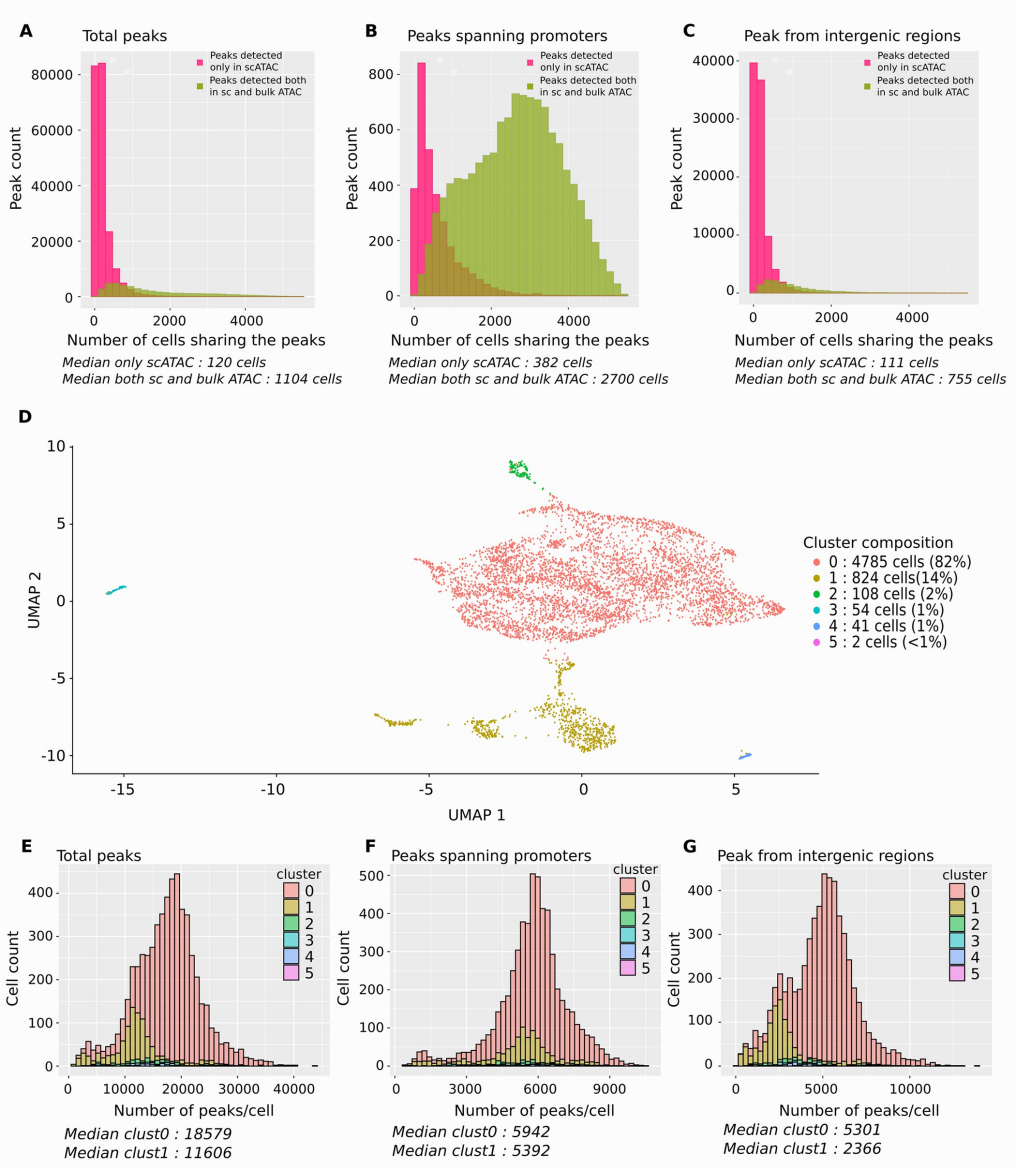
scATAC-seq analysis of chromatin accessibility at 24 h poststimulation. (**A**) Histogram of the distribution of the total number of peaks as a function of cells sharing them. The distribution of the peaks detected only by scATAC-seq are in pink, and those detected both by single-cell and bulk ATAC-seq are in green. The median numbers are indicated below the histogram. Note that the peaks detected by scATAC-seq only are shared by a low number of cells only, while those detected by both methods are present in a large number of cells. (**B**) Histogram of the distribution of the promoter-specific peaks as a function of cells sharing them. The color codes are the same as in (**A**). The peaks detected by both methods are shared by a very large number of cells. (**C**) Histogram of the distribution of the intergenic peaks as a function of cells sharing them. The color codes are the same as in (**A**) and (**B**). The peaks detected by both methods are shared by more cells than those detected only by scATAC-seq, but both numbers are low. (**D**) UMAP visualization of the scATAC-seq data. Two significant groups of cells were identified; a major (82% of the cells) and a minor (14% of all the cells). (**E**) Histogram of the distribution of total number of peaks detected per cell belonging to the large (cluster 0) or small (cluster 1) cluster as seen in (**D**). Note the lower number of peaks in the cells of the small cluster. (**F**) Histogram of the distribution of promoter-specific peaks detected per cell belonging to the large (cluster 0) or small (cluster 1) cluster. Here, the difference between the number of promoter-specific peaks is smaller between the 2 clusters. (**G**) Histogram of the distribution of the intergenic peaks detected per cell belonging to the large (cluster 0) or small (cluster 1) cluster. More intergenic peaks were detected in the large cluster than in the small. scATAC-seq, single-cell ATAC sequencing; UMAP, Uniform Manifold Approximation and Projection.

The unusually high number of accessible gene promoters raised the possibility that this reflects the mixture of several markedly different subpopulations with distinct chromatin profiles. To test this hypothesis, we performed scATAC-seq on a 24-h sample, because the average number of genes expressed per cell and the level of mRNAs were close to maximum at this time point. We used the same number of cells as in the bulk version (5,000 cells). The results are shown on **Fig 5**.

Overall, more than 250,000 accessible sites (peaks) were detected after aggregation of the single-cell data. This number is comparable with the number of peaks detected in each individual donor in the bulk ATAC-seq experiment. Most of these peaks in scATAC-seq were shared by a low number of cells (the median value is only 120 cells sharing a peak), signifying that half of the peaks were present in less than 2.4% of the cells (**Fig 5A**). If we consider only the promoter-spanning insertions, the median number of sharing cells was 382 (**Fig 5B**), whereas the median number of the cells sharing the same intergenic regions peaks was 111 (**Fig 5C**).

When the results of the bulk and single-cell approaches were compared, we found that 99% of the peaks obtained by “bulk” ATAC-seq were also detected by the single-cell approach. In addition, the commonly detected peaks between bulk and single-cell ATAC-seq are also the most shared peaks between cells in the single-cell dataset. The median number of cells that shared one of these peaks was 1,104 (**Fig 5A**). This number is much higher if we consider only the gene promoter-spanning peaks. Each of the 11,570 promoter-spanning peaks detected in “bulk” were also detected by the “single-cell” approach, indicating that these promoters were reproducibly detected as accessible in the cells of each donor. Between 2,000 and 4,000 cells shared about the half of these peaks (the median number of cells was 2,700; **Fig 5B**). Qualitatively similar picture emerged from the comparison of the peaks detected in the intergenic regions by the “bulk” and “single-cell” approaches (**Fig 5C**). The intergenic peaks identified by both the “bulk” and “single-cell” approach were shared by a median number of 755 cells versus the 111 cells for the peaks detected only by the single-cell approach (**Fig 5C**).

scATAC-seq allows to assess the heterogeneity of the cell population based on the chromatin structure. As shown on the UMAP visualization (**Fig 5D**), a major (82%) and a minor (14% of all the cells) group of cells were identified. This is in good agreement with the single-cell mRNA expression analysis, which also identified a minor subpopulation at 24 h (**Fig 2D and 2J**). The minor population cells had lower number of accessible regions (**Fig 5E**). We found about 10% less accessible promoters and 40% less intergenic genomic sites in the minor compared to the major population (**Fig 5F and 5G**).

As shown in **Fig 5F**, the number of promoter-specific peaks detected in most of the cells was close to 6,000. Given that the average number of genes expressed in individual cell at this time point is between 2,000 and 2,500 (**Fig 1B**), this means that there are more promoters available for transcription than the number of genes actually transcribed. In addition, the median number of promoter-specific peaks shared by the same cell is higher than the same number for intergenic peaks (**Fig 5B and 5C**). Our data do not provide precise explanation for the higher number of promoter-spanning peaks shared between the cells. One can only speculate that intergenic regions made accessible are less engaged in functional interactions than promoter-specific regions. As a consequence, they remain accessible for shorter periods. This results in less cells where a particular intergenic region is accessible at the moment of the snapshot.

In order to assess the heterogeneity of the 11 hematopoietic-specific genes, we visualized the aggregated number of ATAC insertions found in each gene in each cell on the same UMAP projection (**Fig 3C**). The results suggest high gene-to-gene and cell-to-cell heterogeneity and only a modest overall correlation between the accessibility and transcript levels. For example, RUNX1 showed the highest expression at 24 h (**Fig 3A**) and appeared as highly accessible in most of the cells (**Fig 3C**). The poorly expressed GATA1 appeared as poorly accessible in the majority of the cells (**Fig 3A and 3C**). By contrast, CBFA2T3, ERG, or SMAD6 appeared to be highly accessible but were poorly expressed (**Fig 3A and 3C**).

It is likely that the observed cell-to-cell heterogeneity of DNA accessibility at the analyzed loci can only partially be explained by the high incidence of failed detections. Our data provided no information on the intrinsic dynamic fluctuations of the chromatin structure. These fluctuations vary from cell to cell and from locus to locus on a time scale of milliseconds for individual nucleosomes [42] to hundreds of seconds for large chromatin domains [43] that is not comparable to the time scale of this study (hours to days). Nevertheless, these fluctuations also increase the heterogeneity of a population detected by a snapshot and produce a broad spectrum of genome configurations in the cells [44]. This heterogeneity certainly contributes to the overall transcriptional heterogeneity observed in the cells during this period.

Overall, the scATAC-seq confirmed the conjecture of a genome-wide chromatin decompaction made on the basis of the “bulk” approach. In addition, it demonstrated the high cell-to-cell heterogeneity of the chromatin structure in the genome of the 24-h cells, suggesting that the global chromatin remodeling itself is a highly dynamic and variable process.

### Transcriptional burst lags behind chromatin decompaction by several hours

The comparison of the chromatin and transcriptional changes shows that the gene transcription burst followed the chromatin decompaction with a delay that exceeds the usual time scale of transcriptional activation. It is a long-standing conundrum of how the chromatin opens to allow TF access to the regulatory sites. One of the possible explanations is that chromatin opening is initiated by specific TFs [35,45]. For this explanation to be correct, TFs must be present before or at the moment of chromatin opening. Therefore, we examined how the changes in the accessibility of gene promoter regions are related to the changes in gene expression, more particularly to the expression of TF-coding genes. Since the joint detection of mRNAs and accessible sites in the same cell is still technically challenging to provide convincing results, we could only compare independently obtained single-cell RNA-seq and ATAC-seq data.

**Fig 6:**
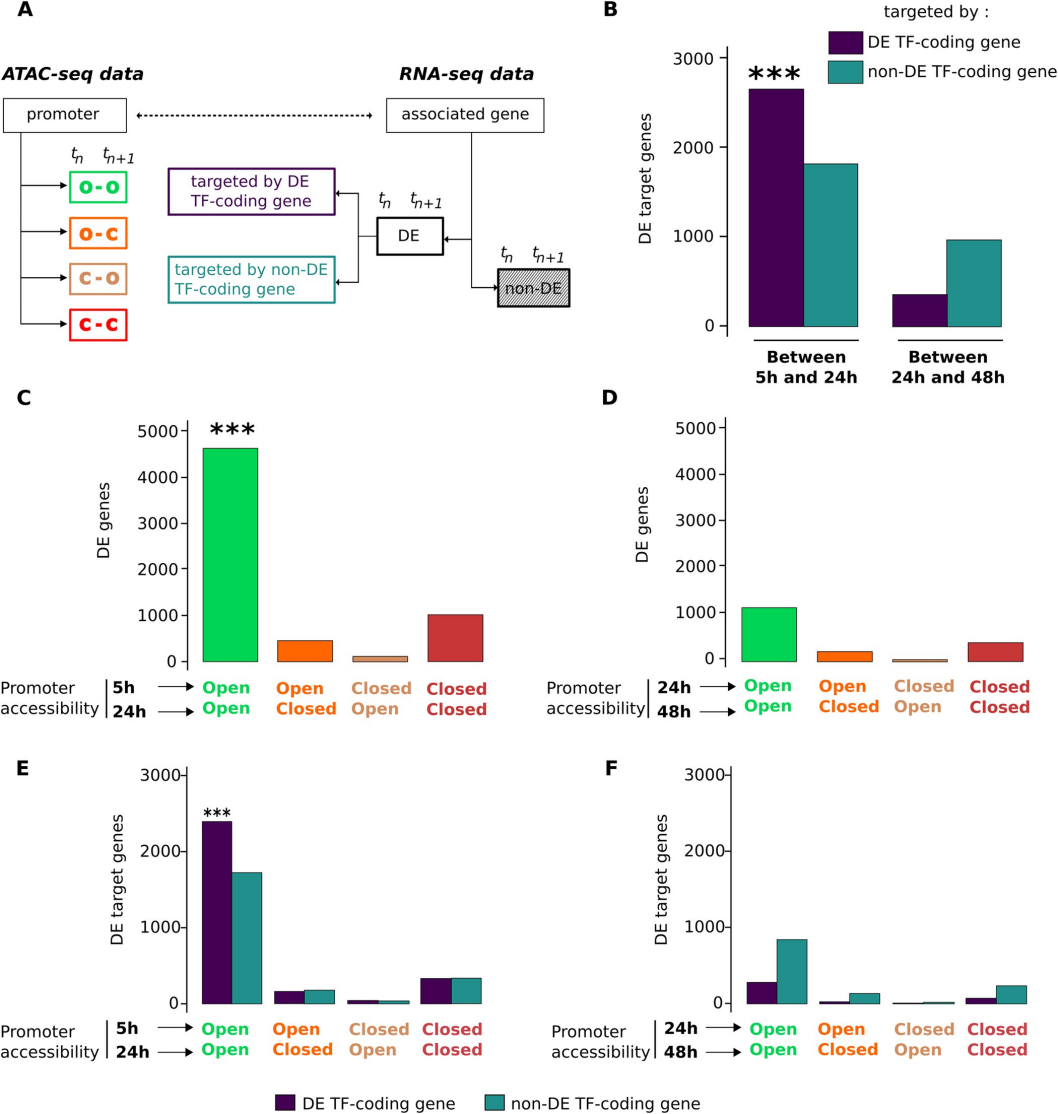
Integration of the ATAC-seq and scRNA-seq data. (**A**) Schematic representation of the classification of the gene promoters on the basis of their accessibility over an interval between 2 time points and genes depending how their transcription changes during the same time interval. The comparison of ATAC-seq and RNA-seq data was done using this classification. (**B**) Enrichment analysis of the DE target genes depending on if they were targeted by DE TF-coding-genes or non-DE TF-coding genes. Left panel: between 5 h and 24 h; right panel: between 24 h and 48 h. The color code identifies the genes targeted by DE TFs or non-DE TFs. As indicated by the asterisks, the genes targeted by DE TFs were overrepresented among the DE genes as determined by two-sided Fisher exact test (*p* = 1.4 × 10^−6^). (**C**, **D**) The total number of DE target genes as a function of the evolution of the promoter accessibility between 5 h and 24 h (**C**) and 24 h and 48 h (**D**). Note that DE genes are significantly associated to the open-open promoter configuration between 5 h and 24 h (indicated by the asterisks; two-sided Fisher exact test: *p* < 10^−4^). (**E**, **F**) The total number of DE target genes as a function of the evolution of the promoter accessibility and as the function of if they are targeted by DE TFs (blue) or non-DE TFs (green) between 5 h and 24 h (**E**) and 24 h and 48 h (**F**) (each category represented on **C**) and (**D**) is divided into two. Note that only the DE genes with open-open promoter between 5 h and 24 h and regulated by DE TFs are significantly overrepresented (indicated by the asterisks; two-sided Fisher exact test: *p* < 2.5 × 10^−7^). (Numerical values available in scRNA-seq repository **GSE156734** used for differential expression “GSE156734_Spread_MARSseq_data_all_filters_20200728.csv”. Numerical values available in bulk ATAC-seq repository **GSE156733** for open/closed promoter information: “GSE156733_peaks_intersection_00h_Xvivo_ann.csv.gz”; “GSE156733_peaks_intersection_05h_MP_ann.csv.gz”; “GSE156733_peaks_intersection_24h_MP_ann.csv.gz”; “GSE156733_peaks_intersection_48h_MP_ann.csv.gz”) DE, differentially expressed; scRNA-seq, single-cell RNA sequencing; TF, transcription factor.

To do this, we expanded the circle of potential players to TFs other than those analyzed above. We explored the expression of every TF-coding gene in our dataset. We sought to determine if there was an association between the changes in the expression of TF-coding genes in general and changes in the expression of their target genes. We assumed that the mRNA levels of TF-coding genes were acceptable surrogates of the TF protein abundance in a cell. If the expression of the TF-coding genes precedes that of their target genes, it is possible that the transcription burst of the target genes is at least partially induced by the TFs. In order to test this hypothetic association, we categorized the genes according to the variation of their mRNA levels. This classification is based on the number of UMIs detected in a cell (see **Materials and methods** section for details). Genes that showed a statistically significant change in the corresponding mRNA level in the 2 donors between 2 time points are referred to as differentially expressed (DE). Between 5 h and 24 h, we found 4,415 DE genes of the total number of 11,248 genes detected. Note that DE genes were essentially up-regulated; only 110 genes were down-regulated. Among those DE genes, we identified 56 TF-coding genes (referred as DE TF-coding genes). Using the Regulatory Circuits resource [46], we found 2,630 DE genes (referred as DE target genes) targeted by at least one of the TFs of DE TF-coding genes (**Fig 6B**). We identified 1,785 DE genes targeted only by one of the 414 non-DE TFs. Using a two-sided Fisher exact test, we demonstrated that there are proportionally more DE genes targeted by DE TF-coding genes than those that are targeted only by non-DE TF coding ones (*p* = 1.4 × 10^−6^) (**Fig 6B**). In other words, if the expression of a TF-coding gene increased, it was frequently paralleled by the increase of the expression of its target genes. However, approximately 40% of the DE target genes increased their transcription without being targeted by a DE TF, suggesting that the TFs are not necessarily required to generate the transcription burst. Using the same approach applied to the period between 24 h and 48 h, only 16 TFs were detected as DE and among them 8 were already classified as such between 5 h and 24 h. No significant difference was found between the proportion of DE genes targeted by the DE and non-DE TF-coding genes (**Fig 6B**). The low level of association between the genes and their TFs and the decrease of the number of gene expression changes suggests that the initial transcription burst observed between 5 h and 24 h came close to the maximum.

We performed GO analysis of the DE TFs and non-DE TFs. A significant fraction of the DE TFs is associated to functionalities related to the hematopoietic system (**S6A and S6C Fig**), such as “regulation of hematopoiesis, myeloid cell differentiation, mononuclear cell differentiation etc.” No such enrichment was found in the group of the non-DE TFs (**S6B and S6D Fig**). The complete list of DE TFs and non-DE TFs together with their target genes are given in **S6 Table.**

As a next step to integrate the gene expression and DNA accessibility observations, we grouped the promoters detected in the ATAC-seq analysis in 4 groups: “open-open”, “open-closed”, “closed-closed” and “closed-open”, depending on how the chromatin around them changed conformation between the 2 time points (**Fig 6A**). We then compared the lists of the genes corresponding to each category of promoters to the lists of DE and non-DE genes. The period between 5 h and 24 h is particularly interesting and important, because most of the changes in gene expression occur at this stage. We found that the promoters of the 74.2% of DE genes were in “open-open” configuration (**Fig 6C**). Hence, their promoters were already accessible 5 h after cell stimulation, long before the burst of transcription. Enrichment analysis showed that this is significantly higher than the proportion of the DE genes in the other categories of promoter configuration (two-sided Fisher exact test: *p* < 10^−4^) (**Fig 6C**). The same analysis performed on data obtained between 24 h and 48 h revealed similar repartition of DE genes among categories of promoter configuration (**Fig 6D**). Particularly, more than 60% of DE genes are associated with the “open-open” promoter configuration. However, during this period, the total number of DE genes is much lower (*n* = 1,849) compared to the 5-h to 24-h period (*n* = 6,230) and statistical tests did not reveal any significant overrepresentation of gene categories (**Fig 6D**).

Finally, we examined how differential expression of TF-coding genes correlated to their target gene transcription and on the DNA accessibility of the target gene’s promoter. To do this, we further divided the category of DE genes with “open-open” promoters into 2 subcategories, depending whether they were targeted by a DE TF-coding or non-DE TF-coding gene (**Fig 6E**). The same subdivision was done for the other categories of promoter configurations also (**Fig 6E**) and for the period of 24 h to 48 h (**Fig 6F**). Between 5 h and 24 h, we found significantly more DE TF-coding genes targeted DE genes in the category with “open-open” chromatin configuration than in all other categories (46%; two-sided Fisher exact test: *p* < 2.5 × 10^−7^) (**Fig 6E**). In comparison, only 33% of the DE target genes were in the non-DE TF-coding gene category with “open-open” chromatin. No significant enrichment was found in the categories between 24 h and 48 h (**Fig 6F**).

Taken together, this complex enrichment analysis points to a rather simple conclusion. The transcription of a gene is more likely potentiated by specific TFs if its promoter was already accessible before the transcription burst. Closed chromatin impedes the action of TFs, even if the expression of this latter increases.

Taken together, the integration of gene expression and chromatin accessibility data revealed the biphasic chronology of chromatin and transcriptional changes in the CD34+ cells. We observed that genome-wide nonspecific chromatin opening that starts before the stimulation of the cells precedes the multilineage-type mixed hyperexpression of the genome. After 48 h, both gene hyperexpression and the number of accessible promoters and extragenic sites started to decrease concomitantly with the emergence of distinct cell populations with particular gene expression patterns.

## Discussion

Progress through a transitional cell state marked by the rise and fall in transcriptional uncertainty and a concomitant rise and fall of cell-to-cell variability was previously reported as a universal feature of cells during the initial phases of the fate commitment process [47]. We show here that the global increase in transcription in CD34+ cells is made possible by the widespread and nonspecific chromatin opening that makes accessible more than 50% of gene promoters in the genome. The process of global chromatin decompaction is initiated before or around the moment the cells are stimulated by the cytokines. By contrast, the burst of transcription that follows the chromatin decompaction by several hours is dependent on the cytokines because without such stimulation, the cells do not grow and die soon. Hence, the transcription burst is made possible but not initiated by the chromatin decompaction. In addition, TFs cannot play a major role in the chromatin opening, because the expression of the genes coding for those factors starts later and remains sporadic. Importantly, the number of gene promoters that become accessible largely exceeds the number of genes that are actually transcribed in each cell (**Figs 1B** and **4B**), raising the question of why some genes become transcribed in a cell while others not. It is hardly possible to explain this observation as a result of a specific and targeted gene activation. Each cell has a different gene expression pattern, and it is highly unlikely that a different specific mechanism is acting in each individual cell. However, if the cell is viewed as a complex system composed of a multitude of interacting components (genes, proteins, small metabolites, etc.), the phenomenon of a global chromatin decompaction followed by a multilineage gene expression burst with a strong stochastic component appears as a manifestation of the state transition typical for dynamical complex systems under stress [48,49]. The rise and fall of the index for critical transitions (I_c_) (**Fig 2F** and **2L**) is an unambiguous indicator that the cells behave as a dynamical complex system close to the transition point between stable states. By creating a permissive chromatin landscape and transiently increasing the transcriptional fluctuations, the nonspecific chromatin opening is likely to be essential to cell fate transition. The preeminent role of the genome reorganization and more particularly of the CTCF protein in the initiation of the transition state is now recognized [50]and further supported by the increased number of the available binding sites seen in our study (**S5 Fig**).

Coherent transcription profiles emerge from this heterogeneous transitory state concomitantly with the gradual chromatin compaction. As a consequence, gene promoters and intergenic sites in the genome become gradually inaccessible again (**Fig 4B**). Some promoters gradually become repressed by chromatin closing, while others are stabilized in an open chromatin configuration. The stabilization of the transcriptome is presumably the consequence of these chromatin changes. Contrary to the initial phase, the role of TFs appears crucial at this stage. Indeed, between 5 h and 24 h, the increase of the transcription of TF-coding genes correlated with the similar increase of their target genes with accessible promoters. No association of the expression of the TF-coding genes is observed with their target genes if their promoters are in “closed” chromatin configuration around the promoter (**Fig 6E**), indicating that chromatin accessibility plays a permissive or gating role for TF action. Since the number of the open promoters is higher at the beginning of the process than the number of expressed genes, a competition for the available TFs among accessible promoters may explain the transcriptional and phenotypic fluctuations observed during this period [26]. These fluctuations cease when the transcriptome is stabilized [26]. The role of TFs may be crucial during the second phase, because their binding may keep the target genes transcribed and prevent the closing of the chromatin. It is worth to remind that TF-coding genes playing a role in the hematopoietic differentiation represent a large fraction of the DE TF-coding genes (**S6 Fig** and **S6 Table**) and that their target sequence motifs are also frequent in the accessible regions (**S5 Fig** and **S5 Table**). It is likely that during the synchronous transcription burst of a large number of genes, the hematopoietic TFs stabilize the open chromatin of their target genes through binding their sequence motifs. In return, this binding stabilizes the TF proteins. Since regulatory sequences of TF-coding genes also bind TFs, the result may be a self-reinforcing network that stimulates the transcription of both the TF-coding and their target genes, as suggested [51].

The proposed scenario of general nonspecific chromatin destabilization followed by a selective repression of the genes is also supported by the observations showing that the inhibition of chromatin compaction using valproic acid (VPA), a histone deacetylase inhibitor, can maintain the multilineage-primed state with promiscuous transcription profile for a long period [26,28,52]. The removal of VPA allows defined transcriptome profiles to be established [28]. Therefore, global chromatin structural changes appear to be causally involved both in the generation of a nonspecific multilineage-primed transcriptional state and the stabilization of the cell fate choice.

The observations reported here represent an example of the general pattern of changes during the process of cell fate choice. Several reports on various cell models converge to conclusions similar to ours. For example, a recent study of human fetal hematopoietic cells demonstrated that extensive epigenetic, but not transcriptional priming of HSC/MPPs, occurs prior to lineage commitment [53]. In another study, monitoring the alterations in the chromatin structure and the nuclear architecture during B cell activation revealed that as quiescent lymphocytes encounter antigens, they rapidly decondense chromatin by spreading nucleosomes from the nuclear matrix to the entire nucleoplasm, decondensing chromatin clusters into mononucleosome fibers, and strengthening their nuclear architecture by creating new CTCF loops and contact domains. The global decompaction and loop formation require Myc, constant energy input, and histone acetylation and are accompanied by an increase in regulatory DNA interactions and gene expression [41]. Studies on hair bulb stem cells also showed that changes in chromatin accessibility precede gene expression changes and lineage commitment [54]. Similarly, the loss of DNA methylation has been shown to be essential for the establishment of chromatin accessibility that determines differential TF binding in neural stem and progenitor cells. Following the differentiation into glial cells, new methylation is acquired to maintain the identity of glial cells by silencing neuronal genes [55]. Furthermore, in human cells, most changes during differentiation arise from dramatic redistributions of repressive H3K9me3 and H3K27me3 marks, which form blocks that significantly expand in differentiated cells [56].

It is of particular importance for further understanding to investigate the process of transcriptome stabilization and the feedback mechanisms that must accompany the emergence of specific gene expression patterns. In this respect, it may be relevant that a dynamic positive feedback loop between permissive chromatin and translational output has been previously reported for embryonic stem and in CD34+ cells [57]. It is noteworthy that many of the genes with the most variable expression that contribute significantly to the specification of the emerging transcription patterns are ribosomal protein (RP)-coding genes (**S2 Table**), thus impacting the process of translation [58]. A high degree of RP expression heterogeneity has already been observed in hematopoietic cells, where a small subset of RPs can discriminate cell types belonging to different hematopoietic lineages [59]. Therefore, it is possible that, in addition to the TF and promoter interactions, a feedback action of the translational output may also contribute to the stabilization of the chromatin. Analogous feedback regulation has been described in ES cells where the translational output directly promotes a permissive chromatin environment, in part by maintaining the levels of unstable euchromatin [57]. Clearly, the selective stabilization of the chromatin is impacted by many more mechanisms, but their respective roles remain to be clarified.

The observations reported here together with other studies bring a new perspective to our understanding of how cell fate commitment is initiated. We propose the following hypothetical scenario. The observed stochastic and highly variable gene expression profile is made possible by the global chromatin decompaction. This can be seen as a rapid but nonspecific response to a substantial and stressful change in the cell’s environment. This reaction is analogous to the physiological stress response whose role is to prepare the organism to meet new and unforeseen circumstances [60]. The mechanisms of the first stage are not yet identified, but explicit and testable hypotheses have been made on their nature [14,15]. The first rapid and nonspecific response is followed by a slower adaptation process that is contingent on the cells own history (cellular memory) and the microenvironmental constraints. The general and nonspecific opening of the chromatin lifts the transcription repression creating the opportunity for the quasi-random activation of genes coding for a large variety of proteins and functional RNAs that were not expressed before. It is likely that in each cell, several concurrent regulatory networks (GRNs) can potentially emerge from the mixed profile. Yet, only 1 GRN will be stabilized at the end of the fate decision process. For example, in our case, each of the CD34+ cells adopt one of the 2 profiles observed. However, the exact nature of the GRN that will emerge from this disorder is not predetermined. The GRN that will prevail in each cell is contingent on the interactions of the cell with the environment including the other cells (extrinsic constraints) and on the cell’s own history recorded by the cellular memory mechanisms (intrinsic constraints). This could be a kind of multistep iterative exploratory trial-and-error process with several potential outcomes. The stochastic fluctuations of molecular interactions within the cell’s nucleus and cytoplasm drive the transitions between the possible GRNs. In a previous paper, using time-lapse microscopy and molecular analysis, we identified “hesitant” cells with this kind of behavior. The GRN that enables the cell to express new functionalities (phenotype) complying better with the constraints in the new microenvironment will be stabilized by feedback mechanisms that reduce the fluctuations. Overall, the process of fate commitment could be viewed as analogous to a continuous iterative process of constrained optimization of the cell phenotype, a kind of “learning process”. This way to conceptualize fate commitment has been theorized long ago [11,12,60–62], and it is compatible by an increasing number of experimental studies [23,26,32,47,63]. The observations reported in this paper shed light on the initial step of this process. We hope that they will contribute to the still unsettled debate on the nature of cell fate commitment and stimulate new experimental studies.

## Materials and methods

### Cell culture

Mononuclear cells were isolated from umbilical cord blood from anonymous healthy donors by density centrifugation using Ficoll (Biocoll, Merck Millipore). Human CD34+ cells were then enriched in the sample by immunomagnetic beads using an AutoMACSpro (Miltenyi Biotec). After collection, enriched CD34+ cells were frozen in a cryopreservation medium containing 90% of fetal bovine serum (Eurobio) and 10% of dimethylsulfoxide (Sigma) and stored in liquid nitrogen.

After thawing, the CD34+ cells were cultured in a 96-well plate in a humidified 5% CO2 incubator at 37°C. Cells were cultured in prestimulation medium made of XVivo (Lonza) supplemented with penicillin/streptomycin (respectively, 100 U/mL and 100 μg/mL; Gibco, Thermo Fisher Scientific), 50 ng/ml h-FLT3-ligand, 25 ng/ml h-SCF, 25 ng/ml h-TPO, and 10 ng/ml h-IL3 (Miltenyi) final concentration.

### Fast ATAC-seq

We used Fast ATAC-seq with minor modifications. This protocol was optimized for blood cells [37]. Prior to transposition, cells were marked with 7AAD and dead cells were removed by FACS (Beckman Coulter). Removing dead cells is an important parameter to ensure clear nucleosome patterns and to improve signal to noise ratio. A total of 5,000 living cells were used at each time point. A 1-step gentle membrane permeabilization and DNA transposition was performed by adding 50 μl of transposition mixture (25 μL TD buffer 2×, 2.5 μL of transposase TDE1 (Illumina), 0.5 μL digitonin 0.1% (Promega), and 22 μL water) to the cell pellets and by incubating at 37°C for 30 min under agitation. Obtained transposed DNA were then purified using MinElute PCR Purification Kit (Qiagen) and preamplified using Nextera barcoded primers (Illumina) and NEBNext High-Fidelity 2xPCR Master Mix (New England Biolabs) for 5 cycles. A quantitative PCR amplification was made on 5 μL of the sample with SYBR Green to determine the number of additional cycles in order to generate libraries with a minimal number of PCR cycles and to limit PCR bias (according to [37]). Appropriate number of PCR cycles were applied on the rest of the preamplified samples. PCR fragments were purified with MinElute PCR Purification Kit (Qiagen) to get rid of unused primers. A supplemental purification step was performed using Ampure beads kit (Beckman Coulter) to size-select DNA fragments ranging between 100 and 700 pb. ATAC-seq libraries were checked for quality using Bioanalyzer (Agilent) prior to sequencing and sequenced in paired-end mode (2 × 50 bp) on the Illumina HiSeq2500 platform.

### Single-cell ATAC-seq

A total of 5,000 living cells collected 24 h after stimulation were used. The experiment and raw data processing/peak annotation was performed by the technical platform of the Institute IMAGINE (https://www.institutimagine.org/en) using the 10X Genomics Chromium technology.

### Single-cell RNA sequencing adapted from MARS-seq

To perform scRNA-seq, we adapted the MARS-seq protocol [64]. CD34+ cells were stained with 7AAD to only work with living cells, and cells were isolated by FACS. Individual cells were sorted into 96-well plates containing 4 μL of lysis buffer with specific barcoded RT primers (final concentration: 0.2% Triton, 0.4 U/μL RNaseOUT (Thermo Fisher Scientific), 400 nM idx_RT_primers). Idx_RT_primers contain a T7 RNA polymerase promoter for further in vitro transcription (IVT), single-cell barcodes for subsequent demultiplexing and UMIs allowing correction for amplification biases. After cell sorting, plates were immediately centrifuged and put into dry ice before storage at −80°C preceding the reverse transcription (RT). To open RNA secondary structure, plates containing single cells were incubated at 72°C for 3 min and immediately put in ice. In each well, 4 μL of RT mix were added (final concentration of RT mix: 20 mM DTT, 2 mM dNTP, 2× First stranded buffer, 5 U/μL Superscript III RT enzyme, 10% (W/V) PEG 8000). PEG8000 was added in the RT mix because it has been shown that it can increase the cDNA yield in scRNA sequencing [65]. ERCC RNA spike-in mix (Thermo Fisher Scientific) was also added to the solution for further amplification quality filtering (dilution 1/40.10e7). The plate was then put into thermocycler (thermocycler program: 42°C– 2 min; 50°C– 50 min; 85°C– 5 min; 4°C–hold).

After first retrotranscription, samples were pooled (see [64]) and ExonucleaseI digestion was performed, followed by 1.2× AMpure beads purification kit (Beckman Coulter) to keep only retrotranscribed single-strand cDNA. Samples were eluted in 17 μL of 10 mM Tris–HCl (pH 7.5). Second strand cDNA synthesis (SSS) using NEBNext mRNA second strand synthesis module kit was then performed (SSS mix: 2 μL 10× SSS buffer, 1 μL SSS enzyme; thermocycler program: 16°C– 150 min; 65°C– 20 min; 4°C–hold). Obtained cDNA was linearly amplified by overnight IVT (HighScribe T7 High Yield RNA synthesis, New England Biolabs) at 37°C under T7 promoter. The product was purified with 1.3× AMpure beads and eluted in 10 μL of 10 mM Tris–HCl, 0.1 mM EDTA. For 3 min, 9 μL of amplified RNA were then enzymatically fragmented with 1 μL of 10× RNA fragmentation reagents (Thermo Fisher Scientific) in 70°C. The fragmentation was stopped with 34 μL of STOP mix (1.2 μL Stop solution, 26.4 μL AMpure beads, 9.8 μL TE), and samples were purified. Differing from original MARS-seq protocol, the second RT was done with primers (P5N6_XXXX) containing random hexamers and specific barcode to distinguish the different plates (i.e., times) (final concentration: 5 mM DTT, 500 μM dNTP, 10 μM P5N6_XXXX, 1× First stranded buffer, 10 U/μL Superscript III RT enzyme, 2 U/μL RNaseOUT; thermocycler program: 25°C– 5 min; 55°C– 20 min; 70°C– 15 min; 4°C–hold). cDNA was purified with 1.2× AMpure beads and eluted in 10 μL.

As for ATAC-seq, the appropriate number of PCR cycles was determined using a fraction of the library with SYBR Green based qPCR as described in [66] (final concentration: 1× Kapa Hifi HotSTart PCR mix, 1× SybrGreen, 0.5 μM mix primer P5.Rd1/P7.Rd2; thermocycler program: 95°C– 3 min– 40 cycles; 98°C– 20 s; 57°C– 30 s; 72°C– 40 s; 72°C– 5 min; 4°C–hold). After PCR amplification, libraries were purified with 0.7× AMpure beads. Libraries were checked for quality, using Bioanalyzer HighSensitivity DNA (Agilent) prior to sequencing. Libraries were finally sequenced in paired-end mode (2 × 50 bp) on Illumina HiSeq2500 platform.

Idx RT primers: TTTTTTTTTTTTTTTTTTTTN = poly-T allowing matching with mRNA poly-A tail, NNNN = 4 bases UMI (randomly generated), XXXXXX = 6 bases cell barcode. The rest of the sequence consists of a PCR adaptor and a T7 promoter sequence for further IVT amplification.

P5N6 XXX: NNNNNN = random hexamer allowing the capture of the fragmented IVT amplified RNA, XXXX = 4 bases “plate barcode”. The rest of the sequence consists of a PCR adaptor.

P5.Rd1/P7.Rd2: P5 and P7 Illumina sequencing adaptors.

### Bioinformatic analysis

#### Single-cell RNA-seq (scRNA-seq) analysis

**Raw data processing**: Cell and plate barcode demultiplexing steps were accomplished under strict selection criteria with the following command:

< cutadapt -q 30 -e 0 -m 30:20—no-trim—no-indels—pair-filter = any >

ERCC mapping was performed using bowtie2 [67] on ERCC known sequences, and regular mapping was performed using STAR [68] on the reference genome version hg19 and aligned reads annotated. After quality filtering, reads and UMIs count per gene and ERCC were calculated for expression analysis.

**Cell and gene filtering**: Chromosome Y was removed from the analysis to avoid unwanted effects, and only protein coding genes were kept for further analysis. Cells with less than 80,000 total reads were removed, as well as cells with more than 10% of reads corresponding to mitochondrial RNA. To reduce undesired effect due to PCR nonlinear amplification, ERCC spikes were used to assess the linearity of amplification. Pearson correlation coefficient was calculated for each cell, and only cells above 0.6 were retained. For each cell remaining, genes were defined as detectable if at least 2 cells contained more than a single UMI (= transcript) and a minimum of 5 reads in total.

**Single-cell clustering and variability analysis**: Clustering analysis was performed with CALISTA [31], a numerically efficient and highly scalable toolbox for end-to-end analysis of single-cell transcriptomic profiles. This approach includes single-cell mRNA counts in a probabilistic distribution function associated with stochastic gene transcriptional bursts and random technical dropout events. In the data preprocessing, we removed cells with more than 95% of zero expression values and then selected the top 200 most informative genes for further analysis. The optimal number of clusters was chosen to be 5 based on the eigengap plot (see [31] for more details). The top “molecular function” GO categories were compared between the clusters using compareCluster function of the Cluster Profiler package [13].

**WGCNA**: We applied WGCNA [69] to the mRNA expression data from each donor separately, to identify modules of genes with similar gene transcriptional dynamics. We excluded genes without any detectable expression in all samples. In implementing WGCNA, we set the soft-thresholding power for a scale-free topology index of 0.9. For each module, we calculated the mean expression of genes by averaging the UMI counts from the 2 donors separately.

**Enrichment analysis**: We obtained a curated collection of TFs to CAGE-defined promoters to gene isoform mapping for a total of 662 human TFs from the Regulatory Circuits resource [46,70]. In our analysis, we used only TF–promoter pairs with moderate confidence scores >0.5. We grouped genes based on whether the relevant TFs demonstrated differential expressions. More specifically, a classification of DE TF was given to any gene in which at least one of its TFs showed a differential expression. Otherwise, a classification of non-DE TF was assigned. A two-sided Fisher exact test was used to perform over- and underrepresentation analysis [71].

**Index for critical transition (I**_**c**_**)**: The I_c_ was calculated from the scRNA-seq-filtered count matrix, as the ratio between the average of gene–gene correlation (Pearson) between all pairs of gene vectors (R(g_n_,g_m_)) and the average of cell–cell correlation between all pairs of cell state vectors (R(c_i_,c_j_)): $I_{C}=R(g_{n},g_{m})/R(c_{i},c_{j})$

The analysis was performed separately for each cluster identified by CALISTA and each donor separately. Only Pearson correlation values higher than 0.70 were considered.

**Uniform Manifold Approximation and Projection (UMAP)**: For each donor, UMI count of the top 200 most varying genes identified by CALISTA were extracted from the scRNA-seq-filtered count matrix. Then, we plotted the cells based on the gene expression in a 2D plot using UMAP representation (package umap v0.2.8.0) and colored the cells either according to their time of collection or their CALISTA cluster affiliation.

#### Bulk ATAC-seq analysis

**Raw data processing**: Tn5 adapters sequences were first trimmed with the following command:

*< cutadapt -q 20 -g "AGATGTGTATAAGAGACAG; max_error_rate = 0*.*1; min_overlap = 10" -A "AGATGTGTATAAGAGACAG; max_error_rate = 0*.*1; min_overlap = 10"—minimum-length 18—times 2—pair-filter = both* >

Genome alignment (hg19) was performed using Bowtie2 with the following parameters:

< bowtie2 -x hg19—no-unal -X 800 >

Only paired-end fragments were kept, considering mapping quality (phred score = 30). Duplicated reads were removed using Picard MarkDuplicates tool. In attempt to not bias the signal recovered after peak calling due to multiple donors, all paired-end files were randomly downsampled to 16 M reads (without disrupting pairs of reads) as regard to the smallest number of reads detected in the cohort (donor1–0 h).

ATAC-seq peaks were then called on those downsampled files using the following:

< macs2 callpeak -f BAMPE -g hs -B—broad—broad-cutoff 0.1—keep-dup all >

In order to retain only significant accessibility peaks across samples, each list of peaks used in advanced analysis has been defined as the intersection between peaks of the 3 donors tested at the same time point.

**Peak annotation**: Peaks were assigned to genomic regions thanks to a homemade script based on the FindOverlap function from the R package “GenomicRanges” [72]. Genomic elements positions (exons, introns, CpG islands, and CTCF) were retrieved from UCSC database (hg19). As for the RNA-seq analysis, promoter regions were retrieved from the online database FANTOM5 [70]. Intergenic category was defined as the exclusion of all other defined categories. No priority has been set across the different genomic elements. Therefore, peaks overlapping several genomic features are counted multiple times, resulting in a total number of peaks across elements exceeding the total number of peaks detected at each time point.

**Peak differential analysis**: DEseq2 tool was used to calculate difference in read count between peaks in 2 consecutive time points [73]. More precisely, the region considered is defined as the interval formed by the union of 2 overlapping peaks at *t2* and *t1*.

**Motif enrichment**: Peak motif enrichment analysis was conducted with the tool “findMotifsGenome.pl” from the HOMER software tool suite [74]. Background file was generated using an autogenerated list of random regions across the genome (hg19). Motifs were scanned using the total length of our peaks by providing the option *<size given>*.

#### s*cATAC-seq analysis and bulk ATAC-seq comparison*

Fastq files generated by Imagine Institute platform were aligned to the hg38 reference genome using Cell Ranger software to obtain count matrix, which was further imported into R (v4.1.2). Using Seurat (v4.1.1) and Signac (v1.6.0) R packages, we integrated the data into a ChromatinAssay object and attributed a genomic annotation using FANTOM5 database for promoters and biomaRt (v2.52.0) for other annotations. Nonstandard chromosomes were removed from the analysis as the number of corresponding peaks was insignificant (<50). TF-IDF normalization followed by singular value decomposition (SVD) were performed on the top features shared by more than 90% of the cells. Dimensions 2 to 30 of LSI reduction were used for cluster analysis. Regarding comparison with the bulk ATAC-seq datasets, aligned reads were shifted from hg38 to hg19 reference genome thanks to Lift Genome Annotations tool. FindOverlap function from GenomicRanges R package (v1.48.0) was used to test the overlap between the accessible regions detected in bulk ATAC-seq and scATAC-seq datasets.

#### ATAC-seq and scRNA-seq combined analysis (accessibility–expression)

**Identification of promoters that have configurational changes**: In an effort to identify promoter regions that are affected (and not affected) by configurational changes of the chromatin, we employed the R Bioconductor package “GenomicRanges” [72]. By comparing the peaks overlapping the promoters between 2 time points (0 h to 5 h, 5h to 24 h, and 24 h to 48 h), we grouped promoters into 4 possible chromatin accessibility configurations: “open-open”, “open-closed”, “closed-open”, and “closed-closed”. We then used the CAGE-defined promoters to gene isoform mapping from the Regulatory Circuits resource [46,70] to identify promoters that overlap with the peaks of ATAC-seq and their corresponding target genes.

**Differential gene expression of single-cell RNA sequencing**: We computed Z-scores for every gene in each of the 2 donors between 2 different time points using the mean and standard deviation of the UMI counts of approximately 100 single cells.


$$Z_{ij}^{t_{2}-t_{1}}=\frac{mean({UMI}_{j}^{t_{2}})-mean({UMI}_{j}^{t_{1}})}{\frac{{\left({\left(sd({UMI}_{j}^{t_{2}})\right)}^{2}+{\left(sd({UMI}_{j}^{t_{1}})\right)}^{2}\right)}^{\frac{1}{2}}}{10}}$$

$Z_{ij}^{t_{2}-t_{1}}$ denotes the Z-score of the expression change of gene *j* in donor *i* between time *t*_2_ and *t*_1_. An average Z-score between the 2 donors was computed and used to identify the set of DE genes. We selected Z-score thresholds of 2 and −2 (i.e., 2 standard deviations of change) to designate up-regulated and down-regulated genes, respectively. Collectively, they represent the set of DE genes.

**Enrichment analysis of combined ATAC-seq and scRNA-seq**: For the combined ATAC- and scRNA-seq analysis, we grouped genes into 8 possible groups based on the chromatin accessibility configurations (i.e., one of the following 4 configurations: “open-open”, “open-closed”, “closed-open”, and “closed-closed”) and whether at least one of their TF-coding genes showed differential expression (i.e., one of the following 2 groups: “DE TF-coding gene” and “non-DE TF-coding gene”). As with the analysis of scRNA-seq data, a gene was assigned to the group “DE TF-coding gene” when at least one of its TFs showed differential expression; otherwise, the gene was classified as “non-DE TF-coding gene”. Note that different isoforms of the same gene can have distinct TSSs that are under the control of different promoters. Thus, a gene might be counted in more than 1 category in the chromatin accessibility configurations. Consequently, the total sum of the genes in the 8 groups as described above might exceed the total number of genes. A two-sided Fisher exact test was used to perform over- and underrepresentation analysis [71].

### Ethics statement

Human cord blood (UCB) was collected from placentas and/or umbilical cords obtained from AP-HP, Hôpital Saint-Louis, Unité de Thérapie Cellulaire, CRB-Banque de Sang de Cordon, Paris, France (Authorization number: AC-2016-2759) or from Centre Hospitalier Sud Francilien, Evry, France, in accordance with international ethical principles and French national law (bioethics law n°2011–814) under declaration N° DC-201-1655 to the French Ministry of Research and Higher Studies.

## Supporting information

S1 Fig Comparative GO enrichment analysis of clusters in both donors. Top GO categories expressed in the cells of the 5 clusters found by CALISTA (p-adj < 0.05). Only genes with pairwise gene–gene correlation scores greater than 0.70 in each cluster were used. Columns correspond to individual clusters (#) from donor1 (d1) and 2 (d2). Numbers of genes associated to each cluster are indicated between parentheses under each cluster number. For GO terms associated statistics and “Entrez” gene IDs, see **S3 Table**. CALISTA, Clustering And Lineage Inference in Single-cell Transcriptional Analysis; GO, gene ontology.
(TIF)

S2 Fig Heat maps representing the single-cell transcript levels in individual cells for donor1 and donor2. Each raw represents a single gene. The gene name abbreviations are indicated on the left of both panels. Each column represents a single cell. Note the heterogenous and low transcript levels detected for each gene, in each cell and at each time point, but with a general tendency to increase between 48 h and 72 h.
(TIF)

S3 Fig Evolution of the ATAC-seq peaks with complex dynamics. Only the peaks that were present at 2 or more time points and display a more complex evolution than the major category of peaks shown on Fig 4D are represented. (**A**) Promoter peaks (blue) and (**B**) intergenic regions (yellow). The number of each profile is indicated on each panel. Note the low number of complex profiles and the low size (number of “read counts”) for both the promoter and intergenic peaks.
(TIF)

S4 Fig Bulk ATAC-seq profiles of the 11 hematopoietic TF-coding genes. Gene names are on the left to each panel. All the time points are shown for each gene. The size of the peaks is indicated in normalized “read counts”. The genes are not drawn to scale. The boxes on the profiles indicate the accessible peaks. Promoter-located peaks are highlighted in red. Note that every gene has accessible promoters but some promoters are only accessible at a single time point.
(TIF)

S5 Fig Selected binding motifs in the accessible chromatin regions. The names, sequence motifs, *p*-values of enrichment, and the frequency compared to the background are shown. CTCF, a major chromatin organizer, shows the highest incidence in the ATAC peaks. The other motifs ate hematopoietic TF-binding sequences. Note that the fraction of motifs accessible remains almost constant over the period examined.
(TIF)

S6 Fig GO analysis of the DE TF and non-DE TF-coding genes. (**A**) and (**C**) show the results for DE TF-coding genes for the time intervals between 5 h and 24 h and 24 h and 48 h, respectively. Note that the significant fraction of the DE TFs is associated to functionalities related to the hematopoietic system. (**B**) and (**D**) show the results for non-DE TF-coding genes for the time intervals between 5 h and 24 h and 24 h and 48 h, respectively. No enrichment for hematopoietic functions is observed. DE, differentially expressed; GO, gene ontology; TF, transcription factor.
(TIF)

S1 Table Quality control summary of MARS-seq data. This table indicates the quality filters used and the number of cells retained for analysis after filtering.
(CSV)

S2 Table Top 200 most variable genes per donor determined by CALISTA and used for cell cluster identification. Note that the list for the 2 donors is almost identical (the first 80 genes are in the same order). The only gene on the list that plays a key role in hematopoietic differentiation is RUNX1.
(CSV)

S3 Table GO terms enrichment statistics per cluster. The table contains the GO terms, descriptions, gene IDs, and *p*-values used to prepare the **S1 Fig.**
(CSV)

S4 Table Bulk ATAC-seq donor-related information. This table indicates the number of unique pairs of sequence reads and the number of peaks detected after downsampling in each donor at each time point.
(CSV)

S5 Table Complete list of motifs used in the enrichment analysis with HOMER. Extensive list of tested motifs and statistics found in peak sequences at each time point. The peak sequences were scanned by HOMER for “known motifs”. Complete HOMER outputs are available on NCBI public repository.
(CSV)

S6 Table Complete list of DE TFs and non-DE TFs together with their target genes. (CSV)

## Abbreviations

CALISTA: Clustering And Lineage Inference in Single-cell Transcriptional Analysis
DE: differentially expressed
GO: gene ontology
GRN: gene regulatory network
HSC: hematopoietic stem cell
IVT: in vitro transcription
MARS-seq: massively parallel single-cell RNA sequencing
RP: ribosomal protein
RT: reverse transcription
scATAC-seq: single-cell ATAC sequencing
scRNA-seq: single-cell RNA sequencing
SVD: singular value decomposition
TF: transcription factor
TFBS: transcription factor binding site
UMAP: Uniform Manifold Approximation and Projection
UMI: unique molecular identifier
VPA: valproic acid
WGCNA: weighted correlation network analysis
