# Supplementary figures and images for: Global genome decompaction leads to stochastic activation of gene expression as a first step toward fate commitment in human hematopoietic cells
Source: PLoS Biol. 2022 Oct 26;20(10):e3001849. doi: 10.1371/journal.pbio.3001849 (PMC9604949; doi:10.1371/journal.pbio.3001849)

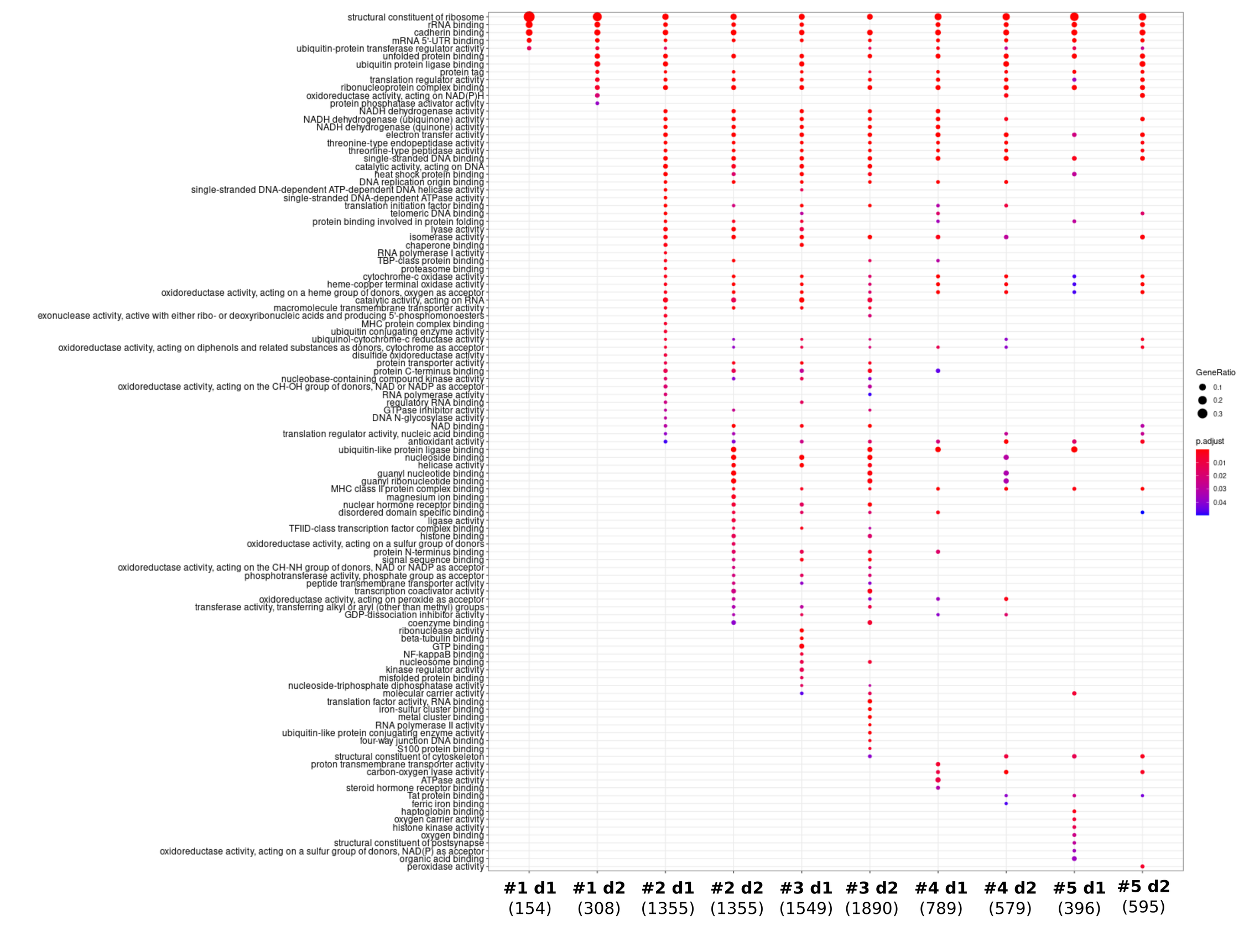

Supplement: S1 Fig — Top GO categories expressed in the cells of the 5 clusters found by CALISTA (p-adj < 0.05). Only genes with pairwise gene–gene correlation scores greater than 0.70 in each cluster were used. Columns correspond to individual clusters (#) from donor1 (d1) and 2 (d2). Numbers of genes associated to each cluster are indicated between parentheses under each cluster number. For GO terms associated statistics and “Entrez” gene IDs, see S3 Table. CALISTA, Clustering And Lineage Inference in Single-cell Transcriptional Analysis; GO, gene ontology. (TIF) [file pbio.3001849.s001.tif]

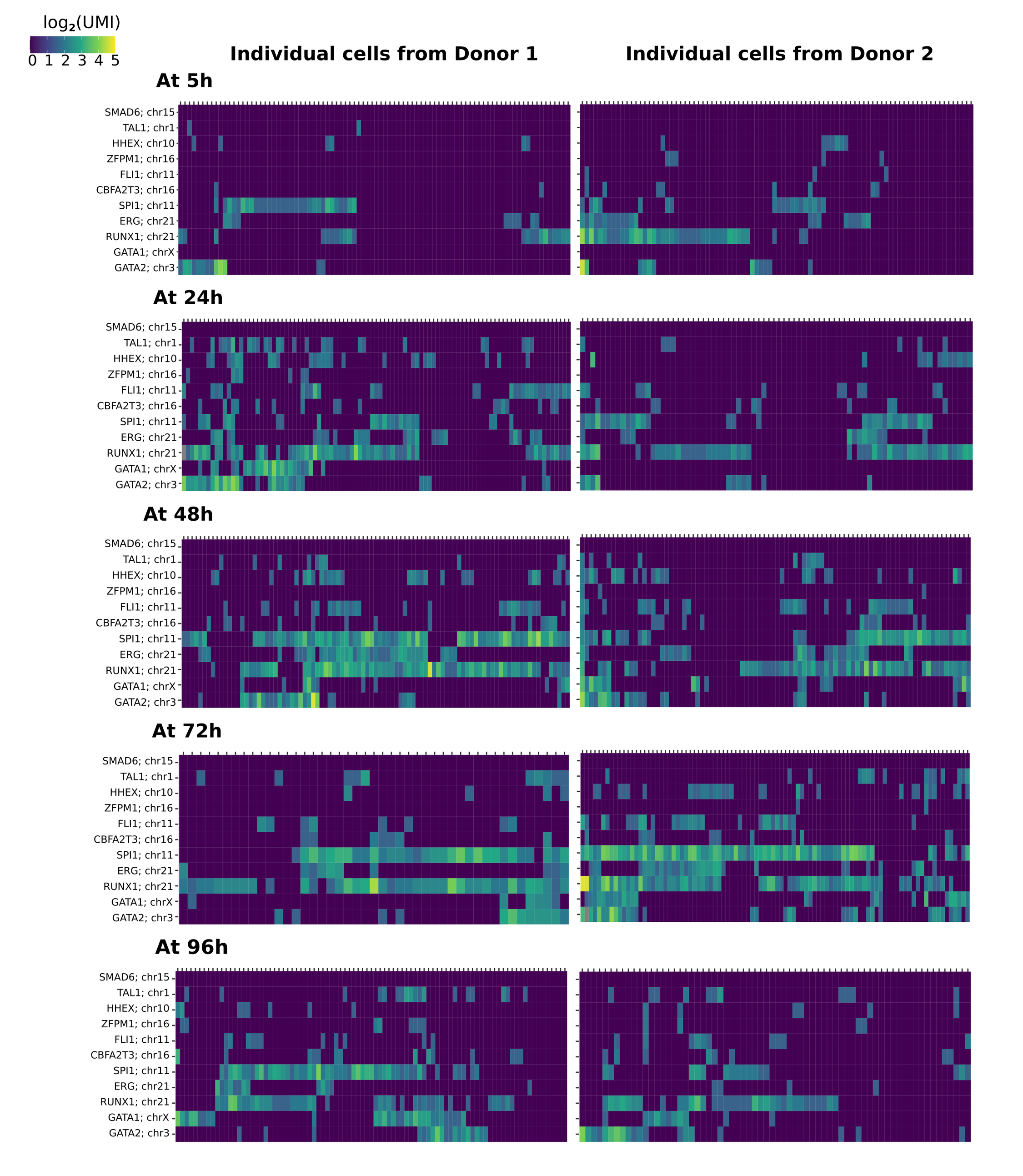

Supplement: S2 Fig — Each raw represents a single gene. The gene name abbreviations are indicated on the left of both panels. Each column represents a single cell. Note the heterogenous and low transcript levels detected for each gene, in each cell and at each time point, but with a general tendency to increase between 48 h and 72 h. (TIF) [file pbio.3001849.s002.tif]

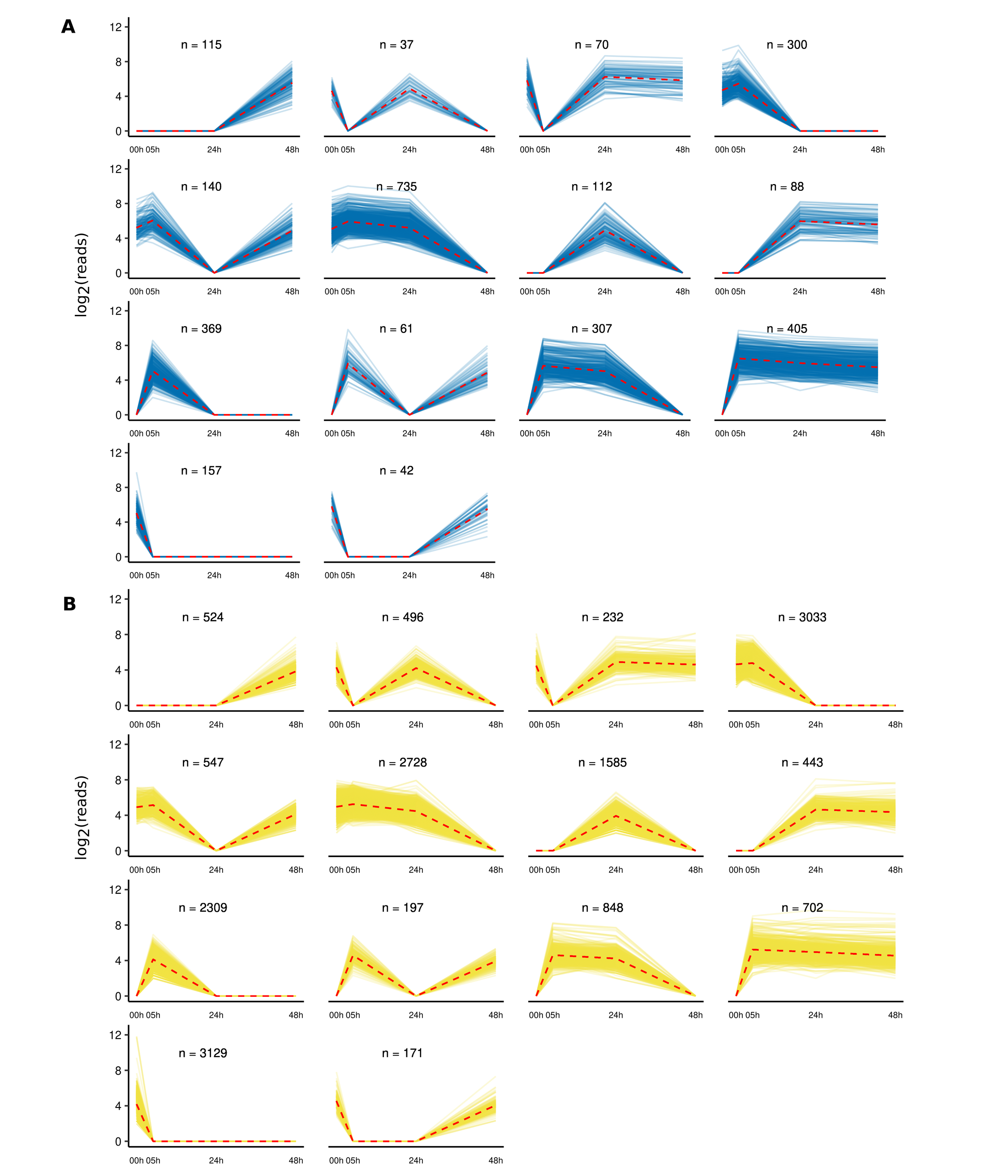

Supplement: S3 Fig — Only the peaks that were present at 2 or more time points and display a more complex evolution than the major category of peaks shown on Fig 4D are represented. (A) Promoter peaks (blue) and (B) intergenic regions (yellow). The number of each profile is indicated on each panel. Note the low number of complex profiles and the low size (number of “read counts”) for both the promoter and intergenic peaks. (TIF) [file pbio.3001849.s003.tif]

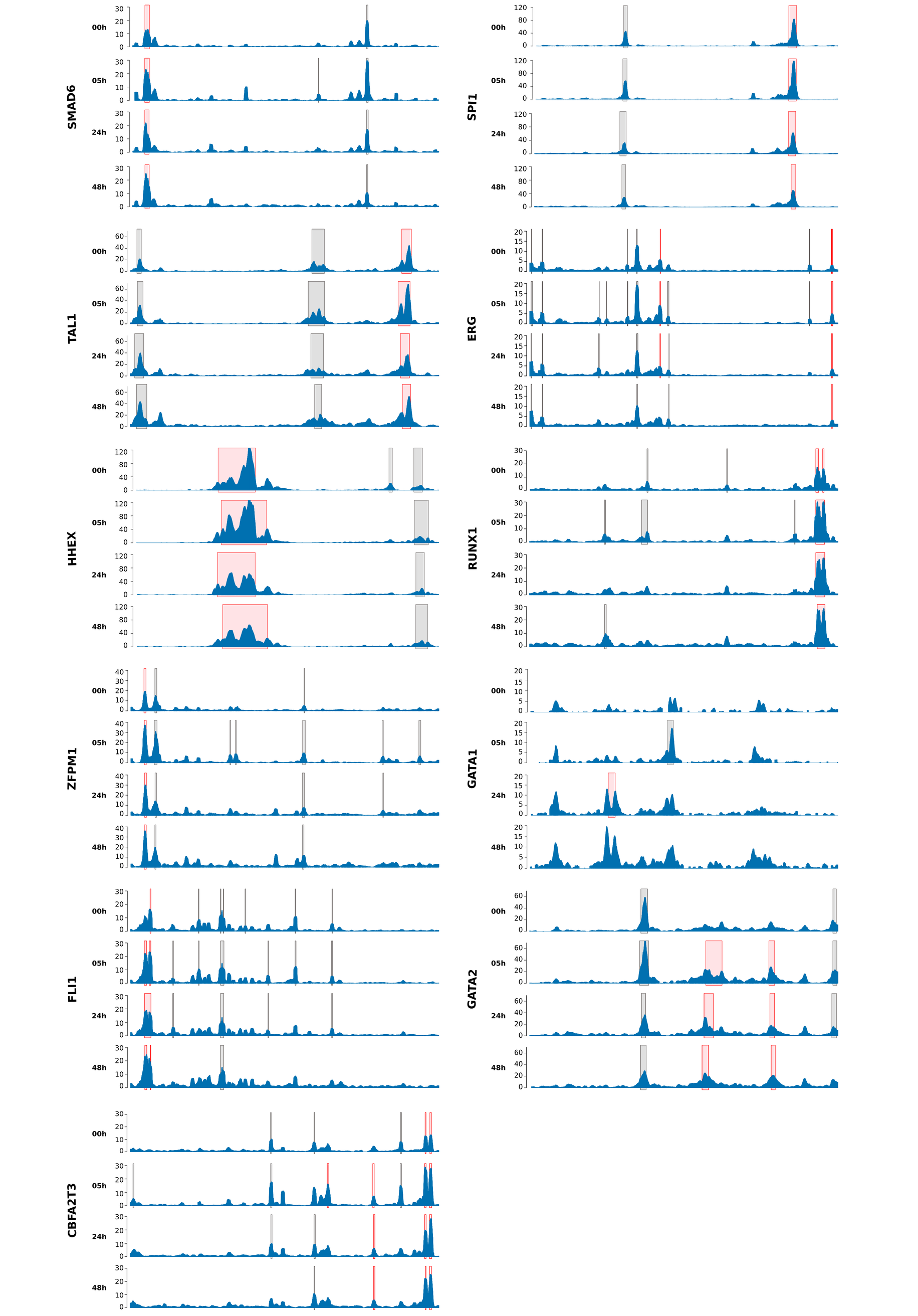

Supplement: S4 Fig — Gene names are on the left to each panel. All the time points are shown for each gene. The size of the peaks is indicated in normalized “read counts”. The genes are not drawn to scale. The boxes on the profiles indicate the accessible peaks. Promoter-located peaks are highlighted in red. Note that every gene has accessible promoters but some promoters are only accessible at a single time point. (TIF) [file pbio.3001849.s004.tif]

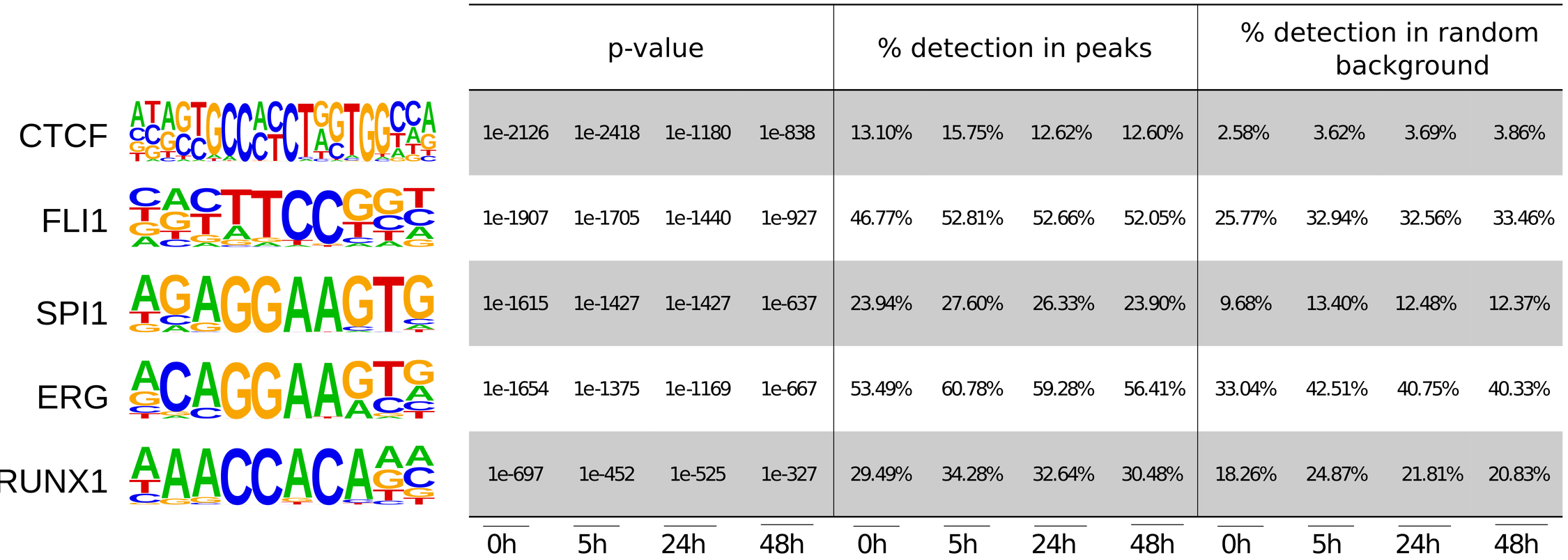

Supplement: S5 Fig — The names, sequence motifs, p-values of enrichment, and the frequency compared to the background are shown. CTCF, a major chromatin organizer, shows the highest incidence in the ATAC peaks. The other motifs ate hematopoietic TF-binding sequences. Note that the fraction of motifs accessible remains almost constant over the period examined. (TIF) [file pbio.3001849.s005.tif]

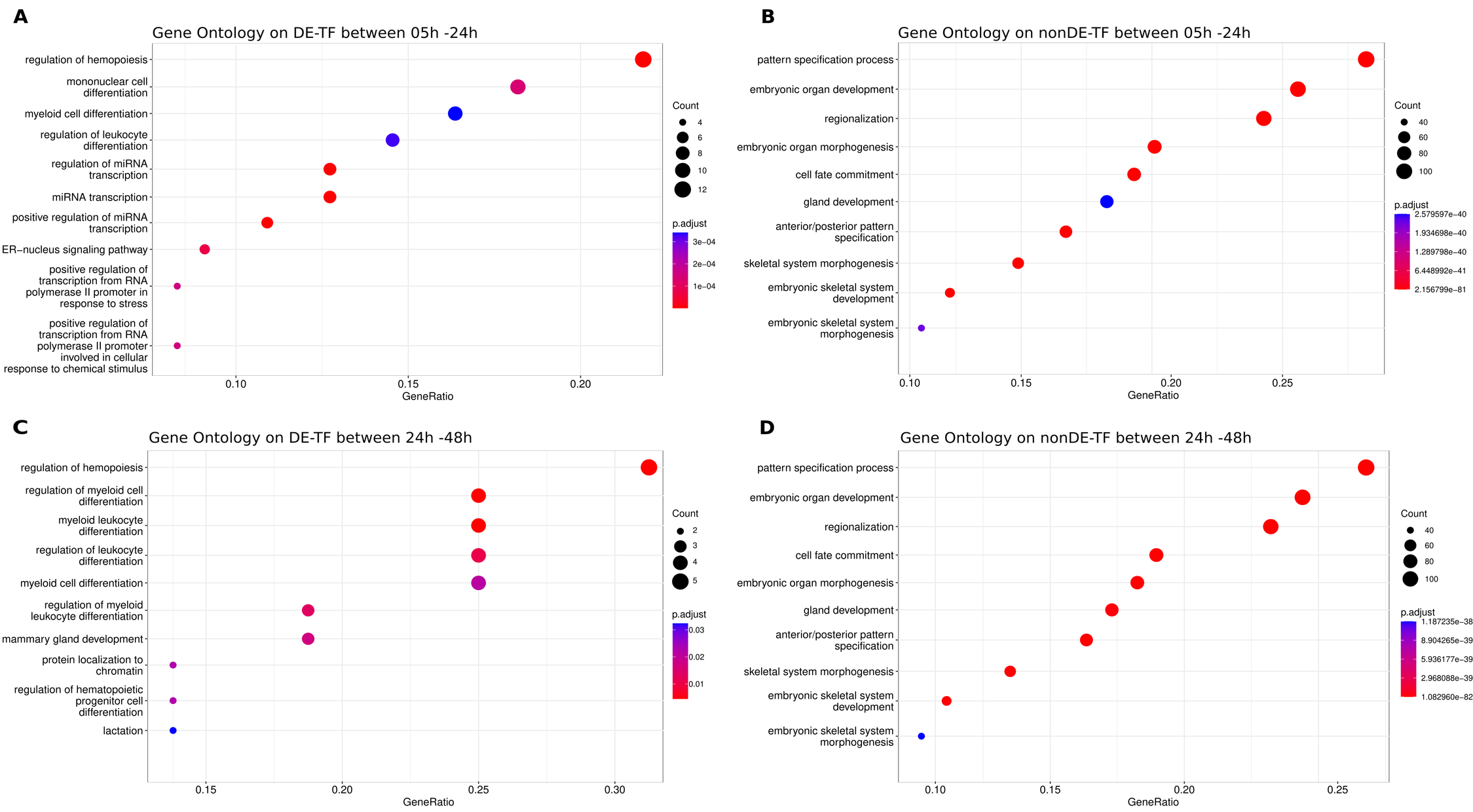

Supplement: S6 Fig — (A) and (C) show the results for DE TF-coding genes for the time intervals between 5 h and 24 h and 24 h and 48 h, respectively. Note that the significant fraction of the DE TFs is associated to functionalities related to the hematopoietic system. (B) and (D) show the results for non-DE TF-coding genes for the time intervals between 5 h and 24 h and 24 h and 48 h, respectively. No enrichment for hematopoietic functions is observed. DE, differentially expressed; GO, gene ontology; TF, transcription factor. (TIF) [file pbio.3001849.s006.tif]
